# Effects of intestinal microbiota on pharmacokinetics of cyclosporine a in rats

**DOI:** 10.3389/fmicb.2022.1032290

**Published:** 2022-11-22

**Authors:** Jinping Zhou, Rui Zhang, Pengpeng Guo, Peixia Li, Xixi Huang, Ye Wei, Chunxiao Yang, Jiali Zhou, Tingyu Yang, Yani Liu, Shaojun Shi

**Affiliations:** ^1^Department of Pharmacy, Union Hospital, Tongji Medical College, Huazhong University of Science and Technology, Wuhan, China; ^2^Union Jiangnan Medical College, Huazhong University of Science and Technology, Wuhan, China

**Keywords:** gut microbiome, fecal microbial transplantation, immunosuppression, cyclosporine A, pharmacokinetics, drug metabolic enzyme and drug transporter

## Abstract

**Background:**

Intestinal microbiota has been confirmed to influencing the pharmacokinetic processes of a variety of oral drugs. However, the pharmacokinetic effects of the gut microbiota on cyclosporine A, a drug with a narrow therapeutic window, remain to be studied.

**Method:**

Twenty-one rats were randomly divided into three groups: (a) control group (CON), (b) antibiotic treatment group (ABT) and (c) fecal microbe transplantation group (FMT). The ABT group was administrated with water containing multiple antibiotics to deplete microorganisms. FMT was with the same treatment, followed by oral administration of conventional rat fecal microorganisms for normalization.

**Result:**

The bioavailability of CSA increased by 155.6% after intestinal microbes were consumed by antibiotics. After intestinal microbiota reconstruction by fecal transplantation, the increased bioavailability was significantly reduced and basically returned to the control group level. Changes in gut microbiota alter the protein expression of CYP3A1, UGT1A1 and P-gp in liver. The expressions of these three proteins in ABT group were significantly lower than those in CON and FMT groups. The relative abundance of *Alloprevolleta* and *Oscillospiraceae UCG 005* was negatively correlated with CSA bioavailability while the relative abundance of *Parasutterella* and *Eubacterium xylanophilum group* was negatively correlated with CSA bioavailability.

**Conclusion:**

Intestinal microbiota affects the pharmacokinetics of CSA by regulating the expression of CYP3A1, UGT1A1 and P-GP.

## Introduction

Cyclosporine A (CSA), a lipophilic molecule, is a powerful immunosuppressive drug used in organ transplantation and autoimmune diseases treatment, with narrow treatment window, mainly metabolized by CYP3A enzyme in liver and excreted by bile (Fahr, 1993). The clinical use of CSA is limited by its side effects, including the nephrotoxic, hepatotoxic, neurotoxic, and cardiotoxic effects (Patocka et al., 2021). Clinical studies indicated that in the renal transplant recipients, concentrations of CSA trough could get lowered safely towards the range of 150–200 ng/ml, added by minimal toxic cyclosporine effects without increased risk for graft rejection (Ragab et al., 2013). Although CSA is traditionally administered at a standard 100 mg dose every day, the resulting exposure can vary greatly between patients and can lead to treatment failure or toxicity. Therapeutic Drug Monitoring (TDM) is a clinical strategy that assesses the response of an individual patient and helps adjust the dosing regimen of CSA to maximize efficacy while minimizing toxicity. However, TDM based dose adjustment could be lagging, and some patients are still at risk of overexposure to or underdose of CSA (Gaies et al., 2019).

Previous studies indicated that age, food, drugs and genetic factors caused variation in CSA pharmacokinetics (Hesselink et al., 2008; Han et al., 2013). Age affects the expression of ABCB1 (encoding the efflux transporter P-GP) gene and the elimination of CSA in the gut and liver (Fanta et al., 2008; Hesselink et al., 2008). Foods help increase bile production, and bile and bile salts appear to be essential for the absorption of cyclosporine (Vonk et al., 1978; Venkataramanan et al., 1986). The most common types of metabolic drug–drug interactions between CSA and other drug are the inhibition and induction of the drug metabolic enzymes (Lake, 1991; Mallat, 1992; Okada et al., 2009). Pharmacogenetics found that CYP450 3A4 and 3A5 variants significantly affect the pharmacokinetics of CSA. (Cascorbi, 2018). However, the role of gut microbiota, being called “the second genome,” in the pharmacokinetics of CSA might be neglected. Studies mentioned that there could be higher risks of graft failure and all-cause mortality in transplant patients with diarrhoea than patients without diarrhoea, but adjusting the dose of immunological agents could improve about 20% of these patients’ graft survival (Kim et al., 2020; Sonambekar et al., 2020). This suggests that microbiota disturbance is a potential factor affecting the pharmacokinetics of cyclosporine A. Therefore, we hope to achieve a more profound and comprehensive pharmacokinetic study to investigate the effect of gut microbiota, so as to realize the best therapeutic effect.

“The human gut microbiome is a complex ecosystem that can mediate the interaction of the human host with their environment,” says Weersma RK (Weersma et al., 2020). The gut microbiota is intricately involved in many of our bodily functions. Pharmacogenomics has been at the forefront of research into the impact of individual genetic background on drug response variability or drug toxicity, and recently the gut microbiota has been recognized as an important player in this respect (Zhu et al., 2010; Doestzada et al., 2018). Manipulating the composition of microbiome is a very attractive way for improving drug efficacy and safety. Gut microbiota affects absorption, enterohepatic recycling, volume of distribution, metabolism and excretion of drugs (Tsunoda et al., 2021; Zhang et al., 2021). Oral drugs might undergo biotransformation by gut microbiota by microbiome located in the intestinal lumen. A study involving 76 different human gut bacteria and 271 oral drugs found that many of them could be chemically modified by microbes *in vivo* (Zimmermann et al., 2019). Digoxin, a cardioside drug, can be inactivated directly by *Eggerthella lenta* (Haiser et al., 2013). In addition, *Faecalibacterium prausnitzii* could convert tacrolimus into a ketone reductor, into which liver microsomes could not metabolize (Lee et al., 2015; Guo et al., 2019). Microbes can also regulate the expression and activity of metabolic enzymes to indirectly influence drug effects (Björkholm et al., 2009). Foley et al. identified that *Clostridia* and *Bacilli* were necessary and sufficient for P-gp induction in the intestinal epithelium in mouse models (Foley et al., 2021). Both *in vitro* and *in vivo* experiments demonstrated that intestinal microbiota could regulate the expression of CYP3A1 (CYP3A4 in human) and P-GP in rats (Hu et al., 2021). CYP3A gene cluster was down-regulated in germ-free (GF) mice, while Cyp4a gene cluster was up-regulated, compared with conventional mice (Selwyn et al., 2016). Ciprofloxacin could reduce liver CYP3A11 expression by inhibiting the production of cholic acid by intestinal microbiota (Toda et al., 2009). The expressions of CYP1A2, CYP2C19, and CYP3A were positively correlated with the alpha diversity of intestinal microbiota (Jarmusch et al., 2020).

At present, the effects of intestinal microbes on the efficacy and toxicity of CSA have been preliminarily reported. Shang et al. ‘s study found that the combination of Xuebijing and CSA was superior to CSA alone in preventing acute graft-versus-host disease by maintaining the intestinal microbial diversity, normalizing the intestinal microorganism and preventing flora disorder (Shang et al., 2022). Another study found that administration of Astragalus and Salvia Miltiorrhiza and fecal microbiota transplantation increased lactic acid-producing probiotics such as *Akkermansia* and *Lactobacillus*, reducing the nephrotoxicity of CSA through the “gut-kidney axis” (Han et al., 2021).

As mentioned above, alteration of the gut microbiota may lead to the changes in the pharmacokinetics of CSA. In this study, antibiotic treatment and fecal transplantation were used to intervene intestinal microbes to examine the role and significance of gut microbiota in the pharmacokinetics of CSA.

## Materials and methods

### Animals

Male Sprague–Dawley (SD) rats (weighing 180–220 g) were purchased from the Laboratory Animal Research Center of Tongji Medical College of Huazhong University of Science and Technology (Wuhan, China), and were given access to a commercial rat chow diet and tap water. The animals were housed, three per cage, and maintained at 22 ± 2°C and 50–60% relative humidity, under a 12 h light–dark cycle. The experiments were initiated after acclimation under these conditions for at least 1 week. The rats were then randomly divided into three groups: ABT group (antibiotic treatment group), CON group (control group), and FMT group (fecal microbiota transplant group; *n* = 14 or 15). The experiments were performed in accordance with the “Guiding Principles in the Use of Animals in Toxicology” adopted by the Society of Toxicology (United States) in July 1989 and revised in March 1999.

### Antibiotic and feces treatment

**Figure 1:**
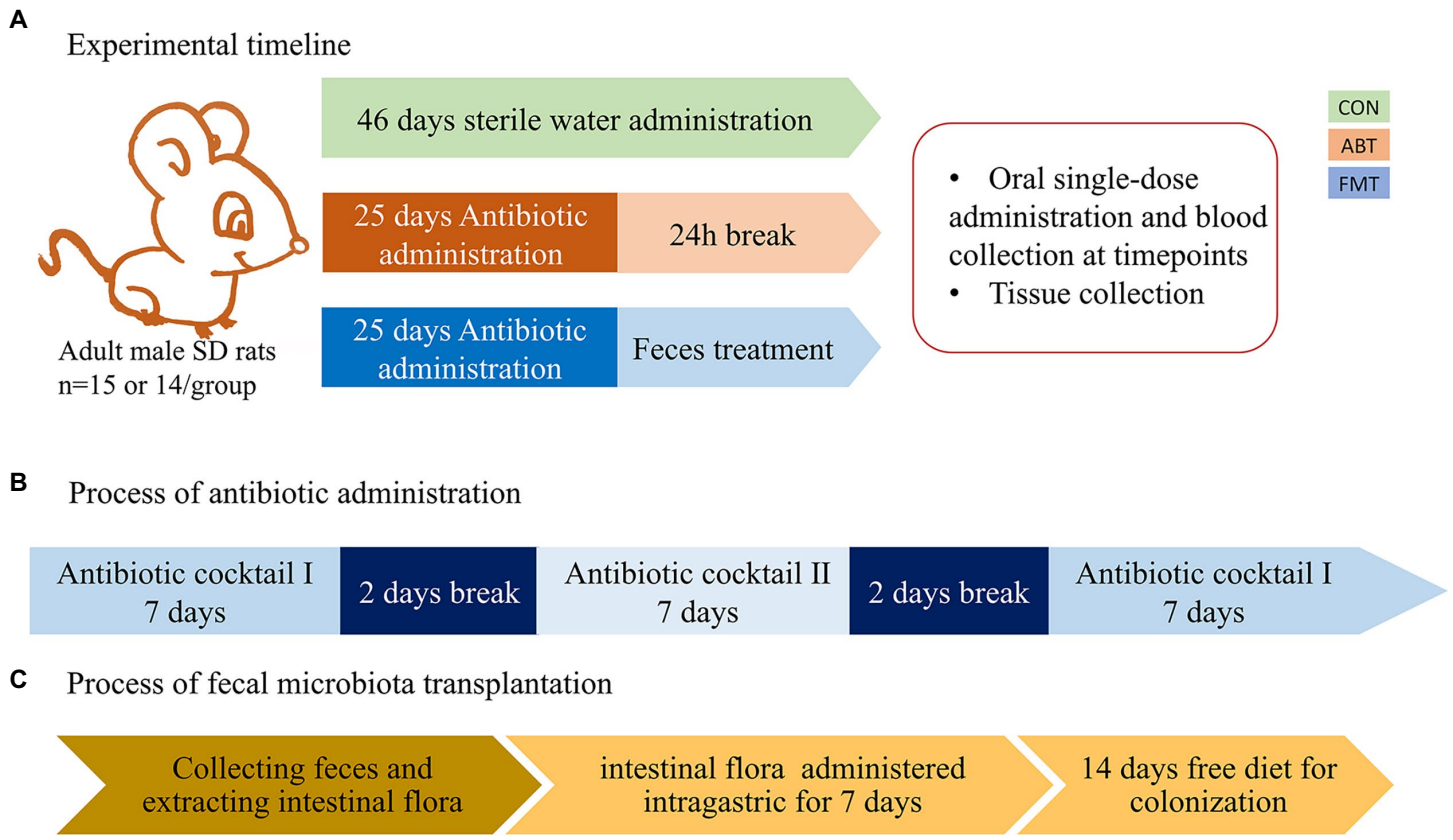
The experimental timeline and the process of antibiotic and feces administration. **(A)** the experimental timeline; **(B)** the process of antibiotic administration, Antibiotic cocktail I consisted of neomycin 1 mg/ml, vancomycin 1 mg/ml, ertapenem 1 mg/ml. Antibiotic cocktail II consisted of clindamycin 1 mg/ml, ambenzyl 1 mg/ml, cefoperazolone 1 mg/ml; **(C)** the process of fecal microbiota transplantation.

Antibiotics were administered for 25 days in the drinking water (Staley et al., 2017). Two kinds of antibiotic cocktail were used in the study. Antibiotic cocktail I consisted of neomycin 1 mg/ml, vancomycin 1 mg/ml, and ertapenem 1 mg/ml. Antibiotic cocktail II consisted of clindamycin 1 mg/ml, ampicillin 1 mg/ml, and cefoperazolone 1 mg/ml. And the solutions were freshly prepared every day. Rats in ABT group and FMT group were administrated with antibiotic cocktails I on days 1 to 7 and 19 to 25. And on days 10 to 16, rats were treated with antibiotic cocktail II. There was a two-day break between antibiotic changes. The feces were collected from control rats and vortex into suspension with buffer phosphate solution (200 mg in 1 ml), followed by centrifugation at 2000 rpm for 5 min to obtain supernatant. The rats in FMT group were treated with the supernatant 2 ml per rat for 7 days, followed by 2 weeks of normal feeding for colonization. The experimental timeline is shown in Figure 1. Rats in CON group were fed sterile water instead of the antibiotic cocktail for 46 days in the same environment.

### Cyclosporine administration and sample collection

There was a 24-h interval after pretreatment to reduce the occurrence of antibiotic-drug interactions. After the 24 h interval, a single dose of CSA (100 mg/kg), dissolved in virgin olive oil, was administered to the rats *via* oral gavage. Whole blood samples were collected at 1, 2, 4, 6, 7, 8, 10, 12, 24, 48 and 72 hours after CSA administration. After the last whole blood sample was collected, the rats were sacrificed with pentobarbital sodium by intraperitoneal injection and dissected to collect tissue and cecum contents. All samples were snap frozen after collected and kept at-80°C until further analysis.

### High performance liquid chromatography detection of cyclosporine in whole blood

The protein precipitation method and high-performance liquid chromatography-mass spectrometry (HPLC–MS) were used to extract and detect CSA in whole blood samples. The method for the determination of CsA was based on our previous developed LC–MS/MS methods (Yang et al., 2018). The linear concentration range of CsA was 5–4,000 ng/ml, and the lower limit of quantification was 5 ng/ml. More details were provided in Supplementary Material.

### Pharmacokinetic analysis

The blood concentration data were analyzed by the non-compartmental method using Drug and Statistics software (DAS, version 3.2.8, Shanghai BioGuider Medicinal Technology Co. Ltd., Shanghai, China). The peak blood concentration (Cmax) and time to reach Cmax (Tmax) of CsA were acquired directly from the concentration-time curve. The elimination rate constant (Kel) was calculated by log-linear regression of the phase-eliminated data. The area under the plasma concentration-time curve (AUC0-t) from time zero to the time of last measured concentration (Clast) was calculated by the linear trapezoidal rule. The AUC zero to infinity (AUC0-∞) was obtained by the addition of AUC0-t and the extrapolated area determined by Clast/Kel. And the terminal half-life (T1/2) was calculated by 0.693/Kel. The mean residence time (MRT) was calculated by AUMC/AUC, where AUMC represented the area under the first moment versus time curve. Apparent clearance (CL/F) was calculated by Dose/AUC0-∞ and the apparent volume of distribution (V/F) was calculated by CL/Kel.

### Microbiota composition

Total genome DNA from samples was extracted using CTAB/SDS method. DNA concentration and purity was monitored on 1% agarose gels. According to the concentration, DNA was diluted to 1 ng/μL using sterile water. 16S rRNA genes of distinct regions (16SV3-V4) were amplified used specific primer with the barcode. All PCR reactions were carried out with Phusion^®^ High-Fidelity PCR Master Mix (New England Biolabs). Mix same volume of 1X loading buffer (contained SYB green) with PCR products and operate electrophoresis on 2% agarose gel for detection. Samples with bright main strip between 400 and 450 bp were chosen for further experiments. PCR products was mixed in equidensity ratios. Then, the mixture of PCR products was purified with Qiagen Gel Extraction Kit (Qiagen, Germany). Sequencing libraries were generated using TruSeq^®^ DNA PCR-Free Sample Preparation Kit (Illumina, United States) following manufacturer’s recommendations and index codes were added. The library quality was assessed on the Qubit^®^ 2.0 Fluorometer (Thermo Scientific) and Agilent Bioanalyzer 2,100 system. At last, the library was sequenced on an Illumina NovaSeq 6,000 platform and 250 bp paired-end reads were generated. More details were provided in Supplementary Material.

### Protein extractions and western blots

Place 20 mg tissue in round-bottom microcentrifuge tubes or Eppendorf tubes，adding 400 μl of ice-cold lysis buffer (with PMSF) and homogenized with an electric homogenizer. Centrifuge for 20 min at 12,000 rpm at 4°C in a microcentrifuge. Gently remove the tubes from the centrifuge and place on ice, aspirate the supernatant, and place in a fresh tube kept on ice. Concentrations of total cellular protein were determined using a BCA assay kit (Beyotime Biotechnology, China). Total protein samples were analyzed by 8% SDS-PAGE gel and transferred to PVDF membranes by electroblotting. Primary antibodies against CYP3A1 (1/5000, ab22733), CYP3A2 (1/5000, ab195627), UGT1A1 (1/6000, ab194697), P-gp (1/6000, ab170904), BSEP (1/3000, ab217532), MRP2 (1/5000, ab203397) and NTCP (1/5000, ab131084) were probed with proteins on the membrane for 3 h at room temperature, and then incubated with goat anti-rabbit secondary antibody (1/10000, ab6721) for 1 h. Bands were detected by enhanced chemiluminescence (ECL) kit (Beyotime Biotechnology, China). The intensity of the bands of interest was analyzed by Image J software (Rawak Software, Inc. Munich, Germany). The gray scale of internal reference protein was normalized for statistical analysis, and the significance was analyzed by one-way variance test and Dunnett test.

### Statistics

T-test, Wilcox rank-sum test, Tukey test, and Kruskal test were performed to analyze whether the differences between groups were significant. Linear discriminant analysis (LDA) was used to reduce the dimension of the data and evaluate the influence of species with significant differences (LDA Score). Zero-mean normalization was performed to normalize the values of relative abundance using the mean and standard deviation (Z Score). Data of pharmacokinetic, including AUC, Cmax and so on, and western blot were expressed as mean ± SD. Spearman’s correlation coefficient to determine the monotonic correlation. The absolute value of Spearman’s correlation coefficient (ρ) reflected the strength of correlation. Meanwhile ρ > 0 meant a positive correlation, and ρ < 0 meant a negative correlation. Statistical analysis was performed using GraphPad Prism 5.0 software (GraphPad Software, Inc., La Jolla, CA, United States) and SPSS (Statistical Product Service Solutions, IBM, United States). The threshold for statistical significance was set at *p* < 0.05.

## Results

### Antibiotic and feces treatment affecting the composition of microbiota in the cecum

**Figure 2:**
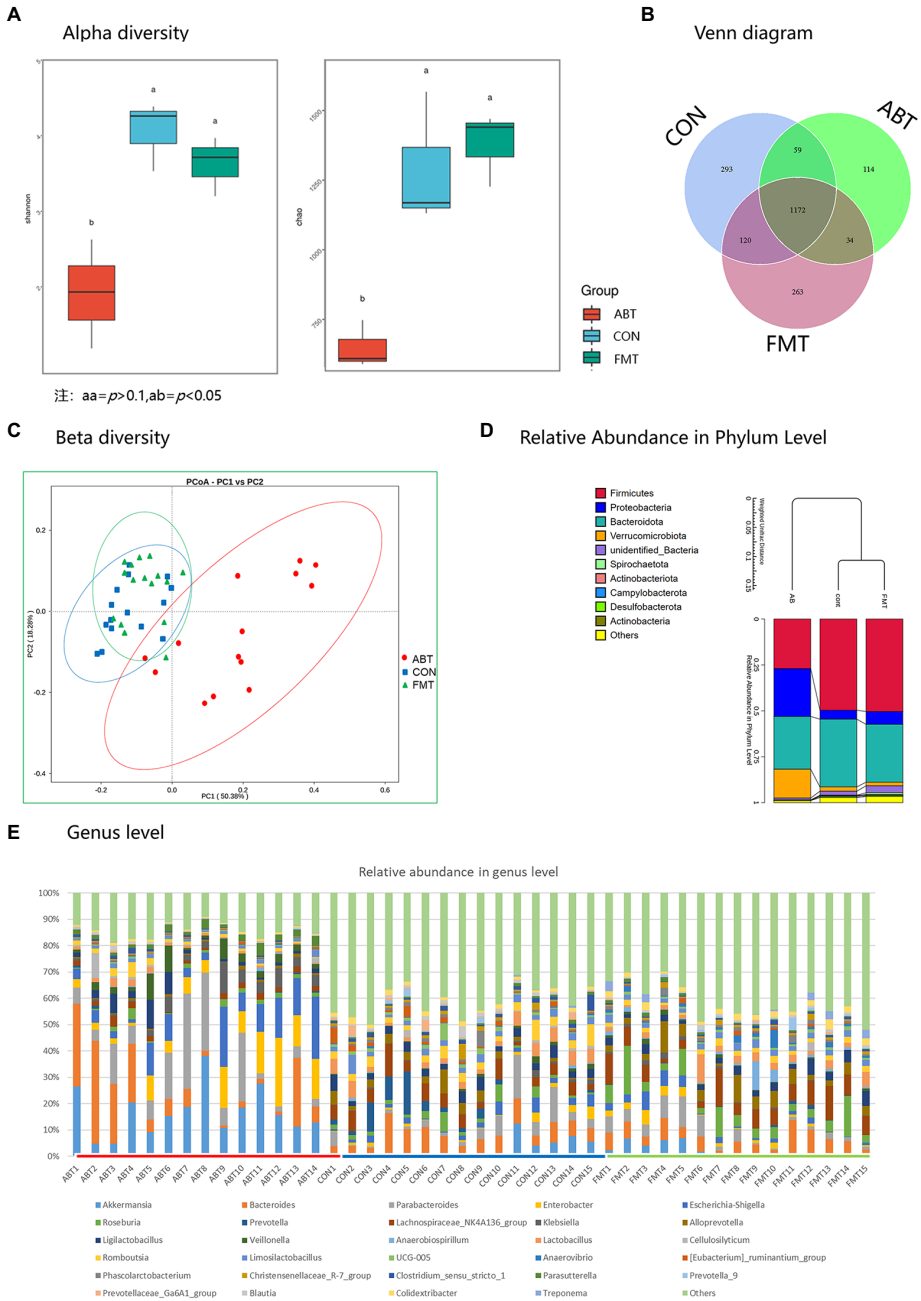
16S rRNA sequencing results. **(A)** is the Shannon and Chao index analysis chart of the three groups of α diversity analysis; **(B)** is a Venn diagram; **(C)** is the β diversity analysis diagram of the three groups; **(D)** is the relative abundance of microbiota at phyla level in each group; **(E)** is the relative abundance of microbiota of each sample at the genus level.

To evaluate the effects of antibiotic treatment and fecal transplantation on the gut microbiota of rats, Sequencing of the bacterial ribosomal RNA (16S rRNA) was performed to compare the bacterial populations on the caecum content. The results revealed that the species richness and diversity were lower after antibiotic treatment than in the CON group, but the depletion was reversed after feces microbiota transplantation (Figure 2A).

The difference in the number of OTU was small (Figure 2B). Moreover, compared with the rats in the ABT, the composition of intestinal microbes of rats in the FMT was more similar to that in the CON group according Figure 2C; Supplementary Figure S1. Results of principal coordinate analysis (PCoA) showed that the species composition of ABT group was significantly different from that of CON group and FMT group (*p* < 0.01, Figure 2C). Differences at phylum and genus levels were also analyzed (Figures 2D,E). At the phylum level, several bacteria were altered by both interventions, with antibiotics causing a reduction of Firmicutes/Bacteroidetes ratio. *Firmicutes* and *Bacteroidetes* were dominant in CON group and FMT group (Figure 2D). However, in ABT group, the relative abundance of *Proteobacteria* and *Verrucobacteria* increased significantly, which became the four major phyla together with *Bacteroidetes* and *Firmicutes*. The comparison at the genus level was based on individual rats (Figure 2E). The relative abundance of top 30 was shown in different colors in the bar chart, and the remaining genus were assigned to others. The proportion of the top 30 was higher in the ABT group, which reflected the composition of bacterial genera in the ABT group was simpler, compared with the other two groups. In ABT group, relative abundance of *Akkermansia*, *Parabacteroides* and *Enterobacter* increased, while the relative abundance of *Lachnospiraceae_NK4A136_group*, *Prevotella Lactobacillus and Alistipes* decreased (Supplementary Figure S2).

**Figure 3:**
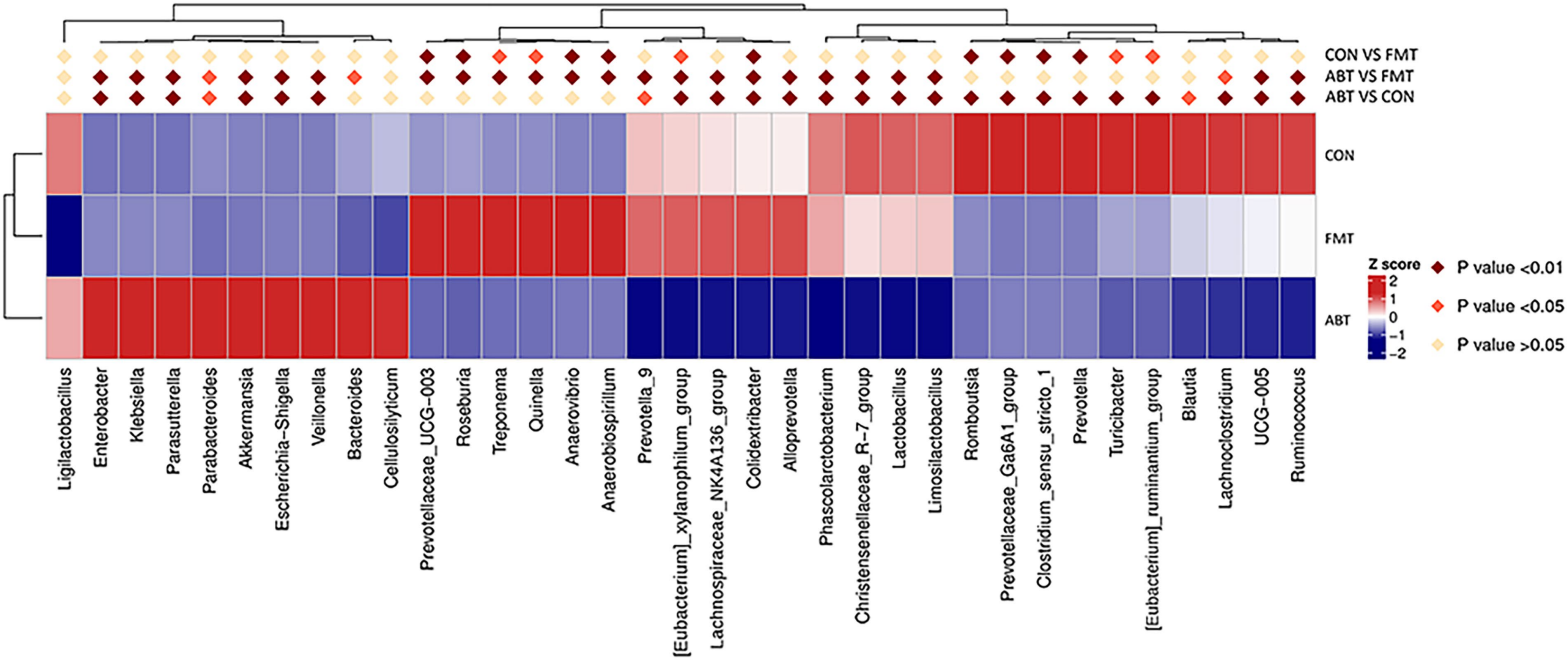
Heatmap of relative abundance for genera with significant difference between groups. Z Score was obtained by zero-mean normalization for each group of values. The colored patches show the *p*-values in the t-test: off-white is *p* > 0.05; orange is 0.05 > *p* > 0.01; dark red is *p* < 0.01.

We compared the relative abundance of each genus by t-test between groups, and *p* < 0.05 was considered to be significant difference (Figure 3). Compared with CON and FMT group, the relative abundance of genera including *Enterobacter*, *Klebsiella*, *Parasutterella*, *Parabacteroides*, *Akkermansia*, *Escherichia-Shigella*, *Veillonella*, *Bacteroides* and *Cellulosilyticum* was significantly increased in ABT group. And the relative abundance of *Prevotella_9*, *[Eubacterium]_xylanophilum_group*, *Lachnospiraceae_NK4A136_group*, *Colidextribacter*, *Alloprevotella*, *Phascolarctobacterium*, *Christensenellaceae_R-7_group*, *Lactobacillus*, *Limosilactobacillus*, *Lachnoclostridium*, *UCG-005*, and *Ruminococcus* was markedly decreased in ABT group, compared with the other two groups. Despite the transplantation of feces from the CON group, the composition of the cecal contents of the FMT group was not the same as that of the CON group. Compared with CON group, the relative abundance of *Prevotella* and *Romboutsia* decreased, while the relative abundance of *Roseburia* and *Anaerovirio* increased in FMT group. This might indicate that compared with *Prevotella* and *Romboutsia, Roseburia* and *Anaerovirio* had stronger colonization ability in the process of feces microbiota transplantation.

### Changes in composition of intestinal microbiota affecting oral bioavailability of CSA

**Figure 4:**
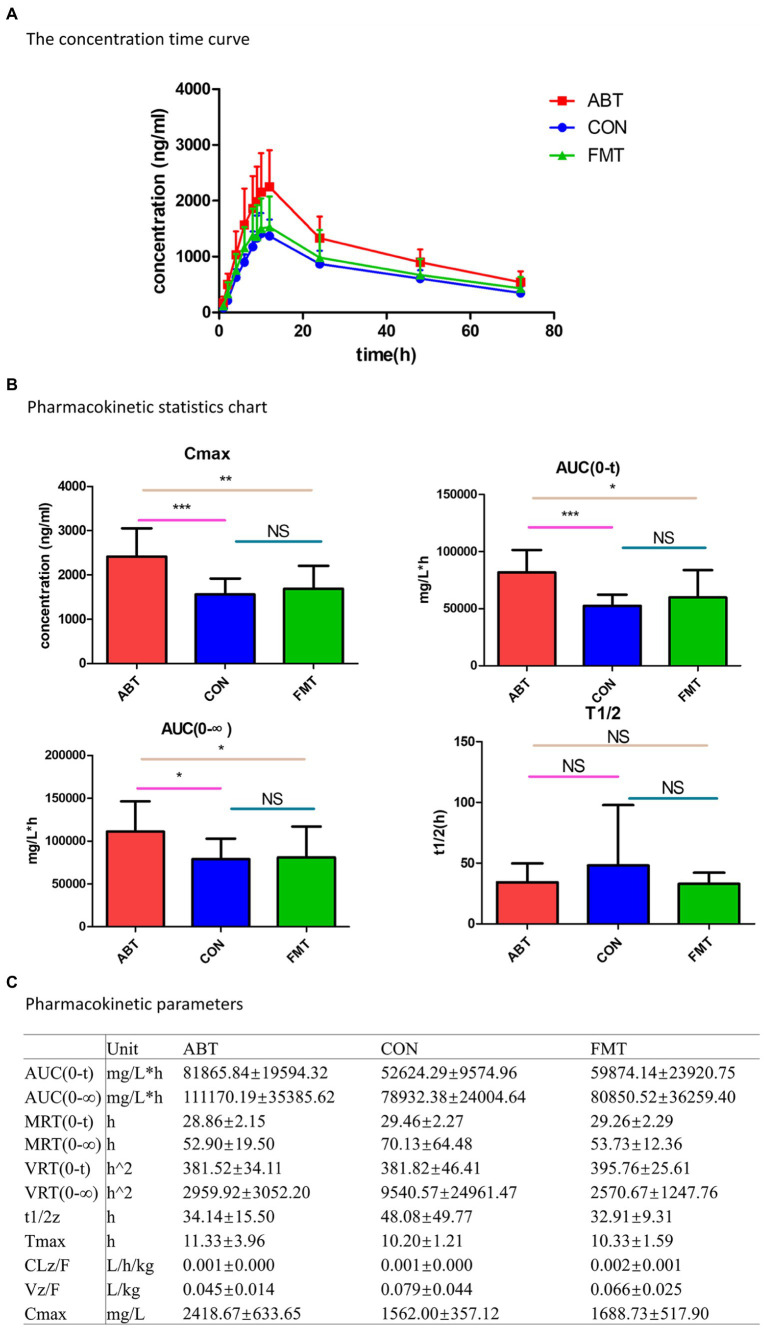
Pharmacokinetic of cyclosporine A. **(A)** Time curve of cyclosporine A plasma concentration; **(B)** Histogram of statistical analysis of cyclosporine A pharmacokinetic parameters; **(C)** is the table of pharmacokinetic parameters of CSA.

After oral administration of CSA, blood concentrations of CSA were determined at each time point in the three groups (Figure 4A). The pharmacokinetic parameters were analyzed by non-atrioventricular model simulation (Figure 4C). Compared to the CON group, the blood concentration of CSA was significantly increased by antibiotic treatment, indicating that the bioavailability of CSA was improved by interfere with intestinal microbial composition. And the pharmacokinetic profiles of CSA in FMT group fell between the ABT group and the CON group. Fecal transplantation could reverse the increased bioavailability of CSA caused by antibiotic treatment.

In addition, statistical analysis of pharmacokinetic parameters showed that AUC_(0-t)_, AUC_(0-∞)_ and C_max_ had statistical differences (*p* < 0.05; Figure 4B). Compared with the CON group, the AUC_(0-T)_, AUC_(0-∞)_ and C_max_ of the ABT group were significantly increased. Antibiotic-induced microbiota depletion led to the increase in AUC_(0-t)_, AUC_(0-∞)_ and C_max_ of CSA by 155.6, 140.8 and 154.8%, respectively. After intestinal microbiota reconstruction by feces microbiota transplantation, the increased AUC_(0-T)_, AUC_(0-∞)_ and C_max_ were significantly reduced and basically returned to the control group level. Half-lives of CSA were not statistically different among the three groups.

### The spearman correlations between the relative abundance of the genera and pharmacokinetic parameters

**Figure 5:**
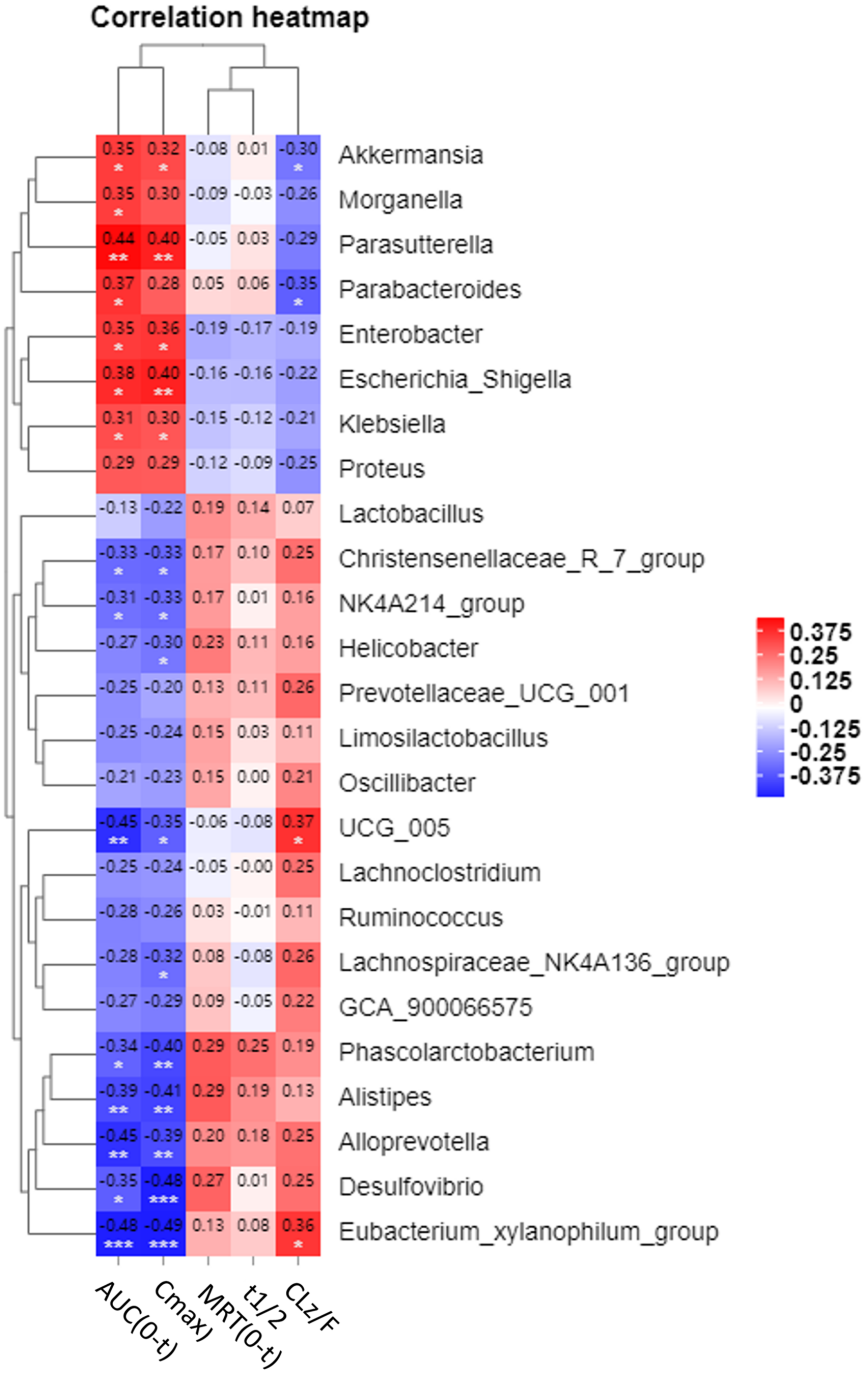
Heatmap of correlations between pharmacokinetic parameters and relative abundance of microbiota at genus level. Values in each square represent Spearman correlation coefficients. * is 0.05 > *p* > 0.01; ** is 0.01 > *p* > 0.005; *** is *p* < 0.005.

After demonstrating that changes in the microbiome occurred in parallel with changes in the systemic absorption of CSA (Figure 3), we compared whether the relative abundance of specific taxa was associated with pharmacokinetic parameters [including AUC_(0-T)_, Cmax, MRD (0-T), t_1/2_ and CLz/F] in three groups (Figure 5; Supplementary Figure S3). Spearman correlation analysis was performed to assess the correlation between relative abundance of genera in Figure 3 and CSA pharmacokinetics (*p* < 0.05 was considered to be relevant). Supplementary Figure S3 showed the scatter plots of 15 genera that were correlated with AUC _(0-T)_. The genera with the|ρ|value exceeding 0.4 were *Alloprevolleta*, *Oscillospiraceae_UCG_005*, *Parasutterella* and *Eubacterium_xylanophilum* (Figure 5; Supplementary Figure S3). The relative abundance of *Akkermansia*, *Morganella*, *Parasutterella*, *Parabacteroides*, *Eeterobacter*, *Escherichia Shigella*, *Klesiella* and *Proteus* positively and significantly correlated with AUC_(0-T)_ and C_max_ of CSA (Figure 5). *Eubacterium Xylanophilum group*, *Desulfovibrio*, *Alloprevotella*, *Alistipes*, *Phascolarctobacterium*, *UCG 005*, *NK4A214 group*, and *Christensenellaceae R^−7^ group* were negatively correlated with AUC_(0-T)_ and C_max_ of CSA (Figure 5).

### Intestinal microbiota altering the level of CYP3As and UGT1A1

**Figure 6:**
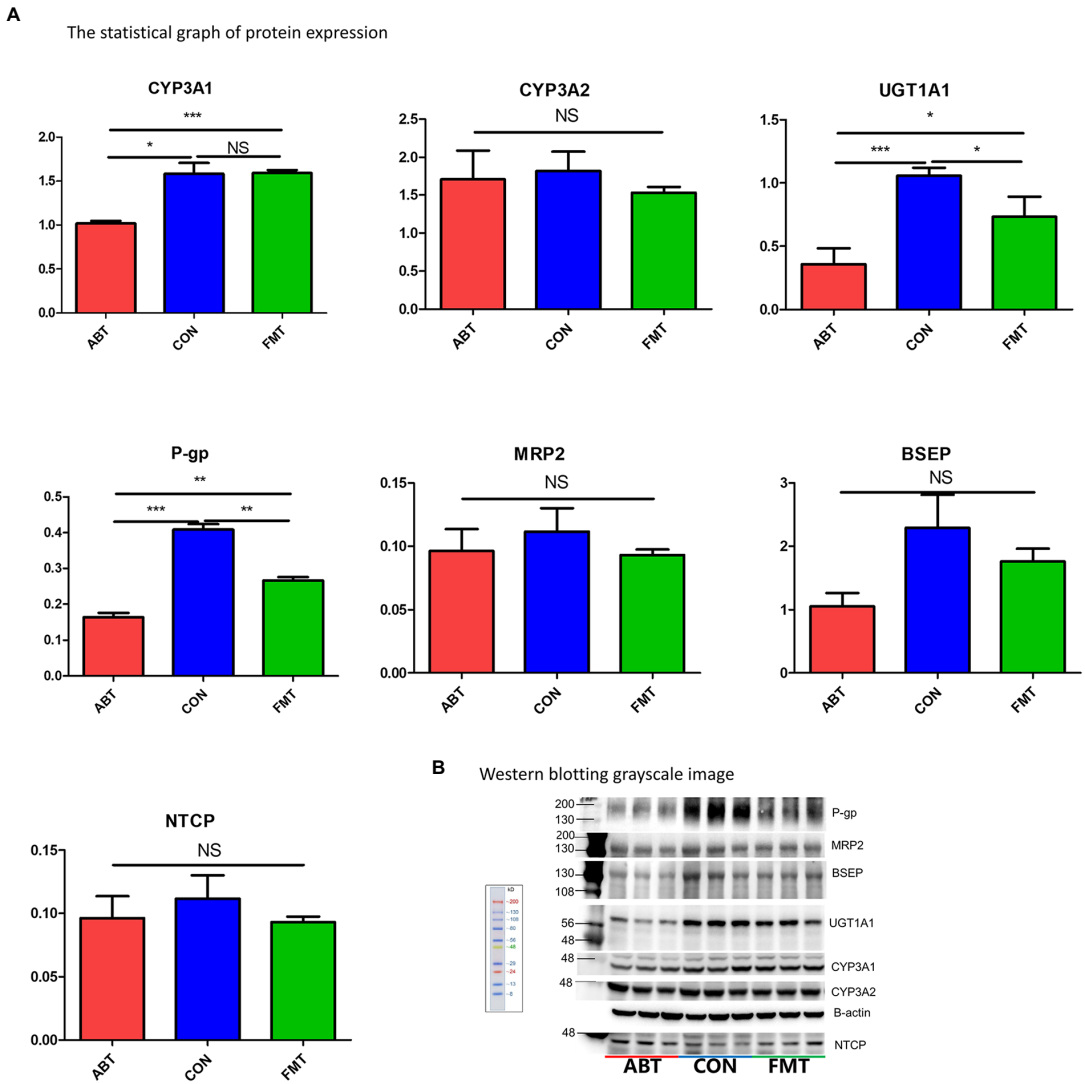
Changes in protein expression. **(A)** is the statistical graph of protein expression. **(B)** is Western blotting grayscale image.

To test whether the changes of intestinal microbiota had an effect on the drug metabolic enzymes (including CYP3A1, CYP3A2, UGT1A1) relevant for the metabolism of CSA in the liver and intestine, western blotting was performed to examine the level of protein expression (Figure 6; Supplementary Figure S4). CYP3A1 and CYP3A2 are the main metabolic enzymes of CSA, which are isomers of human CYP3A4 and CYP3A5 (Martignoni et al., 2006). Compared with the CON group, the expression of CYP3A1 of the ABT group decreased by 35.7% while there was no marked difference between the FMT group and CON group. Hepatic protein expression of UGT1A1, a major two-phase metabolic enzyme of CSA, was decreased by 66.0% in the ABT group and 30.8% in the FMT group compared to the CON group (Figure 6A). Contrary to the results in liver, there was no difference in the expression of UGT1A1 and CYP3A1 in the small intestine among the three groups, and the protein expression of CYP3A2 in the ABT groups was higher than that in the CON group (Supplementary Figure S4). As liver played a more important role in the metabolism of CSA than intestine, the alteration of hepatic metabolic enzymes was consistent with our pharmacokinetic results.

### Intestinal microbiota altering the level of P-gp and MRP2

P-gp and MRP2 were important efflux transporters in the transport of CSA. The hepatic protein expression of P-gp of the ABT group and the FMT group decreased by 59.9 and 34.8% in comparison to the CON group (Figure 6A). Consistent with liver, the protein expression of the efflux transporters P-gp and MRP2 was decreased in the ABT group and reversed in the FMT group (Supplementary Figure S4). Efflux proteins located at the apical membrane, which include P-gp and MRP2, may drive compounds from inside the cell back into the intestinal lumen or biliary excretion, preventing their absorption into blood. In ABT group, the down-regulation of efflux transporters both in the liver and intestine resulted in the increased bioavailability of CSA.

### Changes in intestinal microbiota not altering hepatic expression of BSEP and NTCP but regulating the expression of nuclear receptors FXR and PXR

Previous studies have suggested that intestinal microbiota regulate the bile metabolism (Sayin et al., 2013; Ramírez-Pérez et al., 2017; Ma et al., 2018; Winston and Theriot, 2020). For this reason, we assessed protein expression of two key transporters for the secretion of bile acids from hepatocytes into bile, BSEP and NTCP. Western blotting results showed the protein expression of BSEP and NTCP were not affected by either microbiome targeted intervention (Figure 6). However, compared with CON group, the hepatic level of nuclear protein FXR and PXR was significantly reduced in ABT and FMT group (Supplementary Figure S5). The protein expression of these two nuclear receptors in ABT group was lower than FMT group, but there was no significant difference.

### Antibiotic and feces treatment not changing the physiologic morphology of the liver and proximal colon

**Figure 7:**
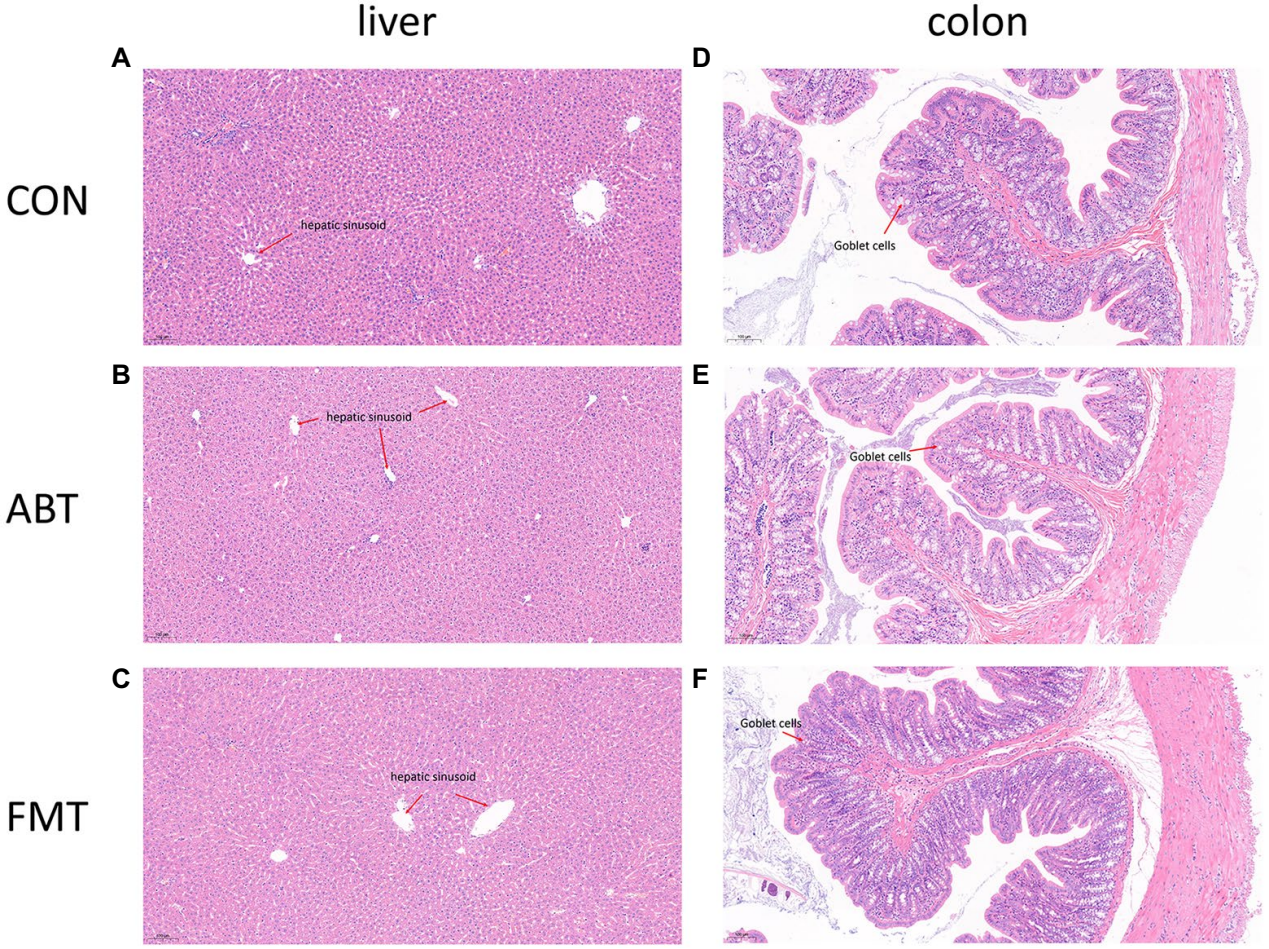
H&E staining of liver and proximal colon sections.

In this study, liver and proximal colon sections were stained with H&E staining to observe the effects of intestinal microbiota intervention on physiologic morphology of liver and proximal colon (Figure 7). Figures 7A–C showed H&E staining of liver sections of the CON group, ABT group, and FMT group, respectively. According to H&E staining results, no significant morphological change was found among the three groups. H&E staining results showed that liver cells were clearly defined, and the cells were evenly and neatly distributed, without obvious aggregation of inflammatory cells among the three groups. Figures 7D–F showed the proximal colon section. The H&E pathological section of rat in CON, ABT and FMT groups did not reveal disappeared crypts and broken structure of colon wall (Figures 7D–F). There was no marked histological damage in livers and colons in three groups.

## Discussion

The gut microbiome affects not only the body’s physiology and pathology but also how the body handles foreign substances, including foods and oral drugs (Jandhyala et al., 2015; Gomaa, 2020; Weersma et al., 2020). Microbiota and drugs interact with each other. Drugs affect the diversity and richness of microbiota, and microbiota also affects the pharmacology and efficacy of drugs. Pharmacomicrobiomics is an emerging field that detects the effect of microbiome alterations on drug pharmacokinetics (ElRakaiby et al., 2014; Aziz et al., 2018; Doestzada et al., 2018; Panebianco et al., 2018; Rowland et al., 2018; Hannachi and Camoin-Jau, 2021). Previous researches suggest that intestinal microbiota had a strong modification effect on the metabolic process of drugs, but the influence of intestinal microbiota changes on drug disposition *in vivo* remains to be further studied (Zimmermann et al., 2019). In this study, antibiotic therapy and feces microbiota transplantation were employed to assess whether the changes in the microbiome affect the pharmacokinetic profiles of CSA. Our results reveal that the bioavailability of CSA was significantly increased after antibiotics depleting gut microbes. Feces microbiota transplantation could reverse the increase in CSA bioavailability caused by microbial depletion. Consequently, the intestinal microbiota played a role in modulating the oral bioavailability of CSA.

According to the results of 16S sequencing, several genera correlated with the pharmacokinetics profile of CSA. *Akkermansia, Parabacteroides, Enterobacter, Escherichia-Shigella, Klebsiella, Parasutterella*, and *Morganella* were positively correlated with the AUC_(0-t)_ and C_max_ of CSA. *Alloprevotella, Oscillospiraceae UCG-005, Phascolarctobacterium, Christensenellaceae R^−7^ group, [Eubacterium] xylanophilum group, Desulfovibrio, Oscillospiraceae NK4A214 group*, and *Alistipes* were negatively correlated with the bioavailability of CSA. This suggested that these specific bacteria may play a role in the alteration of pharmacokinetic profiles of CSA. At the same time, even fed under the same conditions, the intestinal microbiota composition of rats in the same group still had variability, which might be one of the factors leading to individual differences in the pharmacokinetics of CSA.

CYP3A1, CYP3A2, and UGT1A1 are the main metabolic enzymes of CSA in liver (Dupuis et al., 2012; Yu et al., 2016; Hassan et al., 2021). Our results showed that neither of the two microbiome-targeted interventions altered CYP3A2 protein expression, but significantly altered the protein expression of the other two metabolic enzymes in liver. The protein expression of CYP3A1 and UGT1A1 was decreased significantly after antibiotic treatment. Feces microbiota transplantation up-regulated CYP3A1 and UGT1A1 protein expression to near normal level. The changes of microbiome also decreased the protein expression of P-gp, an important efflux drug transporter, in both liver and intestine. The downregulation of CYP3A1, UGT1A1 and P-gp in the ABT group inhibited the metabolism and excretion of CSA in the liver, and reduced the hepatic first-pass effect of CSA. Additionally, in intestine, the protein expression of MRP2 downregulated in ABT group. The decrease in protein expression of P-gp and MRP2 in ABT group could reduce the efflux of CSA in intestine, leading to an increase in the amount of CSA passing through the intestine into the portal vein. However, in contrast to the liver results, there was no difference in the expression of UGT1A1 and CYP3A1 in the small intestine among the three groups, and the protein expression of CYP3A2 in the ABT and FMT groups was higher than that in the CON group. The small intestine played a more critical role in drug transport than in drug metabolism. Therefore, the results of protein expression of drug metabolic enzyme and drug transporters in the small intestine were consistent with our pharmacokinetic results. CYP3A, UGT1A, P-gp, and MRP2 not only played an important role in the absorption and elimination of CSA, but also a large number of oral drugs. We ought to pay attention to the huge potential impact of the gut microbiota on drug personality difference.

How did the gut microbiome alter liver protein expression? In the ABT group, the relative abundance of *Enterobacteria, Akkermansia,* and *Klebsiella* was significantly higher than that of the other two groups. *Akkermansia* is a probiotic deserving research, which could participate in the regulation of intestinal inflammation, but its direct correlation with the expression of drug metabolic enzymes and drug transporters was not reported (Zhai et al., 2019). Nevertheless, studies showed that *Akkermansia* could up-regulate the production of short-chain fat acid (SCFA), which could down-regulate the expression of P-gp in mouse intestine (Zhang et al., 2020; Grajeda-Iglesias et al., 2021). Up-regulation of *Akkermansia* in ABT group could be the reason for down-regulation of P-gp. *Enterobacteria* and *Klebsiella,* gram-negative bacteria, could produce outer membrane vesicles (OMV), which act on TL receptors and altered the expression of CYP3A and P-gp (Gao et al., 2017; Behrouzi et al., 2018). Moreover, the nuclear receptor FXR and PXR in liver down-regulated in ABT group, compared with CON group (Supplementary Figure S5). Metabolites produced by gut microbiota such as SCFA and secondary bile acid could activate or inhibit these two nuclear receptors to regulate the expression of the downstream proteins in liver. Future studies exploring differences in metabolites produced by microbiome, or examining effects of specific bacteria on CSA, are warranted.

Evidence suggested that changes in intestinal microbiota were likely to affect the formation of secondary bile acids (Sayin et al., 2013; Ramírez-Pérez et al., 2017; Ma et al., 2018; Winston and Theriot, 2020). The protein expression level of bile acid-related transporters, NTCP and BSEP, wasn’t altered by gut microbiome significantly in the liver. However, the composition of bile acids in the intestine, liver and serum needs to be determined by further experiments.

In general, intestinal permeability affects the intestinal permeability of oral drugs, especially macromolecule insoluble drugs. The results of H&E staining showed that intervention in the intestinal microbiota did not change the histological morphology of the proximal colon. The high abundance of *Akkermansia* may be one of the reasons for maintaining the intestinal barrier in the ABT group, when the intestinal flora was relatively disorganized. To elucidate whether the intestinal barrier dysfunction was induced by antibiotics, following-up studies on the expression of tight junction proteins are necessary.

However, there were limitations to this experiment. The specific molecular mechanism of bacterial regulation of liver protein expression was not elucidated. Qualitative and quantitative analysis of the metabolites produced by microbes could help us to explain the mechanism in more detail. We overlooked the direct metabolic effect of intestinal microbes on the prototype drug, which might also be a factor influencing the pharmacokinetics of CSA.

In conclusion, this study confirmed that gut microbes could influence the pharmacokinetics of CSA by regulating protein expression of liver drug metabolic enzymes and drug transporters. Patients receiving long-term treatment for anti-rejection may concomitantly take antibiotics along with the CSA. In this case, the use of antibiotics may alter the gut microbiota, resulting in altered metabolism of the CSA. Thus, it could introduce a cause of drug–drug interaction mediated by gut microbiota. Maintaining intestinal microbial stability might be a good way to exert the stabilizing effects of CSA. This study also provided a new perspective for the individualized application of CSA. In addition, fecal microbiota transplantation was expected to be an effective means to improve the poor transplant outcome caused by diarrhea during organ transplantation, for avoiding changes in immunosuppressant drug concentrations caused by fluctuations in microbial levels.

## Data availability statement

The data presented in the study are deposited in the figshare repository, accession number doi: https://doi.org/10.6084/m9.figshare.21260793.

## Ethics statement

The animal study was reviewed and approved by Medical Ethics Committee, Tongji Medical College, Huazhong University of Science and Technology.

## Author contributions

SS, YL, and RZ participated in research design. XH and YW conducted experiments. CY, JZ, TY, and PG contributed new reagents or analytic tools. JZ and RZ performed data analysis and wrote or contributed to the writing of the manuscript.

## Funding

This work was supported by National Natural Science Foundation of China (81874326, 82173902, and 82104274), General Project of Wuhan Health Research Fund (WX21D55), Project of Beijing Medical Award Foundation (YXJL-2018-0095-0068).

## Conflict of interest

The authors declare that the research was conducted in the absence of any commercial or financial relationships that could be construed as a potential conflict of interest.

## Publisher’s note

All claims expressed in this article are solely those of the authors and do not necessarily represent those of their affiliated organizations, or those of the publisher, the editors and the reviewers. Any product that may be evaluated in this article, or claim that may be made by its manufacturer, is not guaranteed or endorsed by the publisher.
