# Supplementary material for: Effects of intestinal microbiota on pharmacokinetics of cyclosporine a in rats
Source: Front Microbiol. 2022 Nov 22;13:1032290. doi: 10.3389/fmicb.2022.1032290 (PMC9723225; doi:10.3389/fmicb.2022.1032290)
Supplement: Supplementary file 1 [file Data_Sheet_1.docx]

**Supplementary information**

1. **Quantification of CsA by HPLC-MS/MS**
   1. **sample preparation**

100μL of whole blood was absorbed into a 1.5mL EP tube, vortexed for 5min with 10μL methanol, 300μL water, 240μL 200ng/mL CsD, and 60μL 0.4mol/L ZnSO_4_. The mixture was centrifuged at 14000rpm for 5min, and the supernatant was collected for analysis.

- 1. **Method of HPLC-MS/MS**

Liquid chromatographic separation and mass spectrometric detection were performed using the AB Sciex API 4000 ™ LC/MS/MS system. Chromatographic separation was performed on an ACE excel 5 C18 column (50 × 2.1 mm). The mobile phase consisted of Acetonitrile (A) and 10 mmol/L ammonium acetate in aqueous solution (B), (containing 0.1% formic acid), at a total flow rate of 0.4 ml/min. The gradient profile for the LC pumps under the final chromatography conditions were as follows: 0 min, 50:50; 1 min, 70:30; 3-5.5 min, 90:10; 6 min, 70:30, 6.5-8 min, 50:50 (A:B, v/v). The injection volume of all samples was 10 μl. The column temperature was set at 40 °C, and the sample tray temperature was maintained at 4 °C. For MS detection, the ESI source was operated in the negative ion mode. High purity nitrogen was used as the sheath (35 arb) and auxiliary (15 arb) gas and high-purity argon was used as the collision gas (1.5 mTorr). The parameters were as follows: spray voltage, 5.5 kV; capillary temperature, 300 °C; scan width for SRM, 0.5 m/z; scan time, 0.2 s. The peak width settings for both Q1 and Q3 were 0.7 m/z. The SRM ion pair transitions and collision energy levels of each component are listed in the Table S1.

Table S1 Parent and product ions of the analytes with individually optimised detection parameters

| Drug | Parent | Product | DP | EP | CE | CXP | retention time (min) |
| --- | --- | --- | --- | --- | --- | --- | --- |
|  | ion[M-H]-(m/z) | ion[M-H]-(m/z) |  |  |  |  |  |
| CSA | 1219.5 | 1203.3 | 56 | 12 | 38 | 23 | 4.15 |
| CSD | 1234.1 | 1217.6 | 40 | 12 | 29 | 25 | 4.21 |

- 1. **Calibration curves**

CSA was dissolved in methanol and prepared into a 1mg/mL stock solution. The stock solution was diluted in methanol gradient to working standard solutions of 40000ng/mL, 20000ng/mL, 10000ng/mL, 5000ng/mL, 1600ng/mL, 400ng/mL, 100ng/mL and 50ng/mL. For analyte identification and quantification, calibration standards were prepared by spiking 10μL of working standard solutions into 100μL of blank rat blood at final concentrations of 5-4000 ng/ml. The solutions for calibration curves were pretreated according to the method in 1.1. Regression line of calibration curve was y=0.000411x+0.0294, R^2^=0.9946.

**1.4 Quantitation of CsA**

The internal standard method was used to quantify the drug concentration in the whole blood of rats at each time point of blood collection. The quantitative results were shown in Table S2.

| Table S2 the whole blood drug concentration of each rat at each time point determined by HPLC-MS/MS | | | | | | | | | | | | |
| --- | --- | --- | --- | --- | --- | --- | --- | --- | --- | --- | --- | --- |
| group | number | Cyclosporine A concentration at different time points(ng/ml) | | | | | | | | | | |
|  |  | 1h | 2h | 4h | 6h | 8h | 9h | 10h | 12h | 24h | 48h | 72h |
| ABT | No1 | 19 | 347 | 571 | 1090 | 2300 | 1890 | 2070 | 1600 | 1190 | 1210 | 826 |
| ABT | No2 | 41.8 | 392 | 639 | 960 | 1230 | 1690 | 1770 | 2210 | 1380 | 963 | 703 |
| ABT | No3 | 222 | 660 | 1430 | 1740 | 2030 | 2240 | 2460 | 2680 | 1510 | 1120 | 483 |
| ABT | No4 | 300 | 709 | 2010 | 3380 | 3250 | 3330 | 3430 | 3390 | 1770 | 998 | 767 |
| ABT | No5 | 183 | 625 | 1310 | 2190 | 2180 | 2010 | 1860 | 1800 | 1110 | 1030 | 605 |
| ABT | No6 | 74.5 | 283 | 1030 | 1310 | 1370 | 1460 | 1620 | 2060 | 1250 | 1010 | 636 |
| ABT | No7 | 55.2 | 292 | 1250 | 1740 | 1830 | 1970 | 2440 | 2020 | 890 | 619 | 365 |
| ABT | No8 | 145 | 287 | 559 | 966 | 1160 | 1030 | 1290 | 1450 | 1550 | 610 | 360 |
| ABT | No9 | 109 | 366 | 647 | 1170 | 1550 | 1620 | 1600 | 1930 | 884 | 643 | 273 |
| ABT | No10 | 391 | 354 | 965 | 1460 | 2280 | 3090 | 3590 | 3050 | 1210 | 978 | 796 |
| ABT | No11 | 164 | 317 | 540 | 745 | 1120 | 1180 | 1120 | 953 | 610 | 472 | 194 |
| ABT | No12 | 230 | 685 | 868 | 1370 | 1480 | 1720 | 2400 | 2780 | 1950 | 870 | 606 |
| ABT | No13 | 243 | 785 | 1150 | 1520 | 1950 | 2390 | 2110 | 2230 | 1160 | 690 | 344 |
| ABT | No14 | 266 | 677 | 1380 | 1940 | 2440 | 2200 | 2580 | 2830 | 1690 | 1200 | 537 |
| ABT | No15 | 191 | 726 | 1180 | 1950 | 1770 | 2020 | 1970 | 2720 | 1830 | 1040 | 610 |
| CON | No1 | 51.1 | 262 | 737 | 845 | 902 | 1020 | 1100 | 1390 | 1000 | 777 | 466 |
| CON | No2 | 106 | 232 | 702 | 994 | 1090 | 1090 | 973 | 1030 | 742 | 635 | 429 |
| CON | No3 | 31.9 | 124 | 465 | 977 | 1450 | 1260 | 1590 | 1410 | 770 | 515 | 279 |
| CON | No4 | 150 | 236 | 408 | 725 | 849 | 830 | 870 | 1060 | 757 | 605 | 344 |
| CON | No5 | 22.4 | 68.8 | 451 | 820 | 1090 | 1360 | 1150 | 1090 | 642 | 401 | 128 |
| CON | No6 | 35.6 | 154 | 476 | 879 | 890 | 879 | 1120 | 1040 | 665 | 513 | 218 |
| CON | No7 | 64.4 | 149 | 641 | 820 | 1110 | 1650 | 1550 | 1250 | 391 | 365 | 336 |
| CON | No8 | 57.2 | 175 | 715 | 907 | 1240 | 1350 | 1820 | 1690 | 898 | 608 | 201 |
| CON | No9 | 80.8 | 81 | 672 | 1080 | 1210 | 1110 | 1660 | 950 | 614 | 589 | 465 |
| CON | No10 | 97.6 | 296 | 657 | 753 | 1060 | 1630 | 1930 | 1780 | 1060 | 818 | 314 |
| CON | No11 | 78.8 | 257 | 590 | 919 | 1340 | 1410 | 1400 | 1270 | 970 | 877 | 503 |
| CON | No12 | 191 | 292 | 821 | 930 | 1140 | 1660 | 1680 | 1500 | 1240 | 431 | 283 |
| CON | No13 | 88.8 | 324 | 734 | 900 | 1060 | 1250 | 1240 | 1690 | 1040 | 599 | 478 |
| CON | No14 | 96.6 | 244 | 427 | 681 | 1200 | 1060 | 1070 | 1540 | 1110 | 732 | 421 |
| CON | No15 | 55.2 | 292 | 890 | 1250 | 1970 | 2440 | 2020 | 1830 | 1140 | 619 | 365 |
| FMT | No1 | 220 | 446 | 1300 | 1810 | 2210 | 2270 | 2380 | 2220 | 1390 | 866 | 639 |
| FMT | No2 | 93.3 | 214 | 660 | 901 | 1290 | 1150 | 1340 | 1210 | 703 | 418 | 372 |
| FMT | No3 | 116 | 432 | 733 | 1270 | 1930 | 1520 | 1800 | 1680 | 1270 | 851 | 495 |
| FMT | No4 | 77.2 | 228 | 837 | 1450 | 1120 | 1390 | 1570 | 2010 | 1230 | 769 | 581 |
| FMT | No5 | 22.9 | 135 | 577 | 760 | 813 | 803 | 845 | 1020 | 489 | 370 | 253 |
| FMT | No6 | 117 | 160 | 465 | 621 | 872 | 744 | 655 | 1330 | 512 | 308 | 275 |
| FMT | No7 | 67.3 | 203 | 615 | 918 | 906 | 848 | 1000 | 983 | 748 | 436 | 245 |
| FMT | No8 | 67.1 | 105 | 369 | 637 | 870 | 886 | 915 | 1190 | 1060 | 726 | 419 |
| FMT | No9 | 120 | 471 | 636 | 1090 | 1290 | 1380 | 1760 | 1220 | 214 | 329 | 95.5 |
| FMT | No10 | 96.2 | 237 | 851 | 1400 | 1160 | 1210 | 1661 | 1500 | 868 | 746 | 355 |
| FMT | No11 | 103 | 179 | 341 | 819 | 1040 | 879 | 1030 | 803 | 664 | 527 | 365 |
| FMT | No12 | 37.1 | 590 | 775 | 1360 | 1300 | 1140 | 1640 | 2100 | 1260 | 929 | 659 |
| FMT | No13 | 256 | 728 | 783 | 1520 | 1920 | 2380 | 2310 | 2510 | 2160 | 1220 | 625 |
| FMT | No14 | 219 | 591 | 1200 | 1380 | 1720 | 1480 | 1520 | 988 | 646 | 515 | 248 |
| FMT | No15 | 202 | 428 | 1190 | 1480 | 2140 | 2340 | 2150 | 2180 | 1510 | 1090 | 864 |

1. **Details of the bacterial 16S rRNA gene sequence**
   1. **Paired-end reads assembly and quality control**

**2.1.1 Data split**

Paired-end reads was assigned to samples based on their unique barcode and truncated by cutting off the barcode and primer sequence.

2.1.2 **Sequence assembly**

Paired-end reads were merged using FLASH (V1.2.7, <http://ccb.jhu.edu/software/FLASH/> ) ^[1]^, a very fast and accurate analysis tool, which was designed to merge paired-end reads when at least some of the reads overlap the read generated from the opposite end of the same DNA fragment, and the splicing sequences were called raw tags.

**2.1.3 Data Filtration**

Quality filtering on the raw tags were performed under specific filtering conditions to obtain the high-quality clean tags ^[2]^ according to the QIIME (V1.9.1, <http://qiime.org/scripts/split_libraries_fastq.html>) ^[3]^ quality controlled process.

**2.1.4 Chimera removal**

The tags were compared with the reference database (Silva database (16S) <https://www.arb-silva.de/> ) using UCHIME Algorithm (<http://www.drive5.com/usearch/manual/uchime_algo.html>) ^[4]^ to detect chimera sequences, and then the chimera sequences were removed ^[5]^. Then the Effective Tags finally obtained.

**2.2. OTU cluster and Species annotation**

**2.2.1 OTU Production**

Sequences analysis were performed by Uparse software (Uparse v7.0. 1001，<http://drive5.com/uparse/>) ^[6]^. Sequences with ≥97% similarity were assigned to the same OTUs. Representative sequence for each OTU was screened for further annotation.

**2.2.2 Species annotation**

16S: For each representative sequence, the Silva Database (<http://www.arb-silva.de/>) ^[7]^ was used based on Mothur algorithm.

**2.2.3 Phylogenetic relationship Construction**

In order to study phylogenetic relationship of different OTUs, and the difference of the dominant species in different samples(groups), multiple sequence alignment were conducted using the MUSCLE software (Version 3.8.31, <http://www.drive5.com/muscle/>) ^[8]^.

**2.2.4 Data Normalization**

OTUs abundance information were normalized using a standard of sequence number corresponding to the sample with the least sequences. Subsequent analysis of alpha diversity and beta diversity were all performed basing on this output normalized data.

**2.3. Alpha Diversity**

Alpha diversity is applied in analyzing complexity of species diversity for a sample through 6 indices, including Observed-species, Chao1, Shannon, Simpson, ACE, Good-coverage. All this indices in our samples were calculated with QIIME (Version 1.7.0) and displayed with R software (Version 2. 15.3).

Two indices were selected to identify Community richness:

Chao -the Chao1 estimator (<http://www.mothur.org/wiki/Chao>);

ACE -the ACE estimator (<http://www.mothur.org/wiki/Ace>);

Two indices were used to identify Community diversity:

Shannon - the Shannon index (<http://www.mothur.org/wiki/Shannon>);

Simpson - the Simpson index (<http://www.mothur.org/wiki/Simpson>);

One indice to characterized Sequencing depth:

Coverage - the Good’s coverage (<http://www.mothur.org/wiki/Coverage>)

**2.4. Beta Diversity**

Beta diversity analysis was used to evaluate differences of samples in species complexity, Beta diversity on both weighted and unweighted unifrac were calculated by QIIME software (Version 1.9. 1).

Cluster analysis was preceded by principal component analysis (PCA), which was applied to reduce the dimension of the original variables using the ade4 package and ggplot2 package in R software (Version 2. 15.3).

Principal Coordinate Analysis (PCoA) was performed to get principal coordinates and visualize from complex, multidimensional data. A distance matrix of weighted or unweighted unifrac among samples obtained before was transformed to a new set of orthogonal axes, by which the maximum variation factor is demonstrated by first principal coordinate, and the second maximum one by the second principal coordinate, and so on. PCoA analysis was displayed by ade4 package and ggplot2 package in R software (Version 2. 15.3).

Unweighted Pair-group Method with Arithmetic Means (UPGMA) Clustering was performed as a type of hierarchical clustering method to interpret the distance matrix using average linkage and was conducted by QIIME software (Version).

**2.5. Prime Sequences**

| name | Linker primer sequence |
| --- | --- |
| 341F | 5’-CCTAYGGGRBGCASCAG-3’ |
| 806R | 5’-GGACTACNNGGGTATCTAAT-3’ |

**Reference**

[1] Magoč T, Salzberg S L. FLASH: fast length adjustment of short reads to improve genome

assemblies. Bioinformatics 27.21 (2011): 2957-2963.

[2] Bokulich, Nicholas A., et al. Quality-filtering vastly improves diversity estimates fromIlluminaamplicon sequencing. Nature methods 10.1 (2013): 57-59.

[3] Caporaso, J. Gregory, et al. QIIME allows analysis of high-throughput community sequencing data. Nature methods 7.5 (2010): 335-336.

[4] Edgar, Robert C., et al. UCHIME improves sensitivity and speed of chimera detection. Bioinformatics 27.16 (2011): 2194-2200.

[5] Haas, Brian J., et al. Chimeric 16S rRNA sequence formation and detection in Sanger and

454-pyrosequenced PCR amplicons.Genome research 21.3 (2011): 494-504.

[6] Edgar, Robert C. UPARSE: highly accurate OTU sequences from microbial amplicon reads. Nature methods 10.10 (2013): 996-998.

[7] Quast C, Pruesse E, et al. The SILVA ribosomal RNA gene database project: improved data processing and web-based tools[J]. Nucl. Acids Res. (2013): D590-D596.

[8] Edgar R C. MUSCLE: multiple sequence alignment with high accuracy and high throughput. Nucleic acids research32.5(2004): 1792-1797.


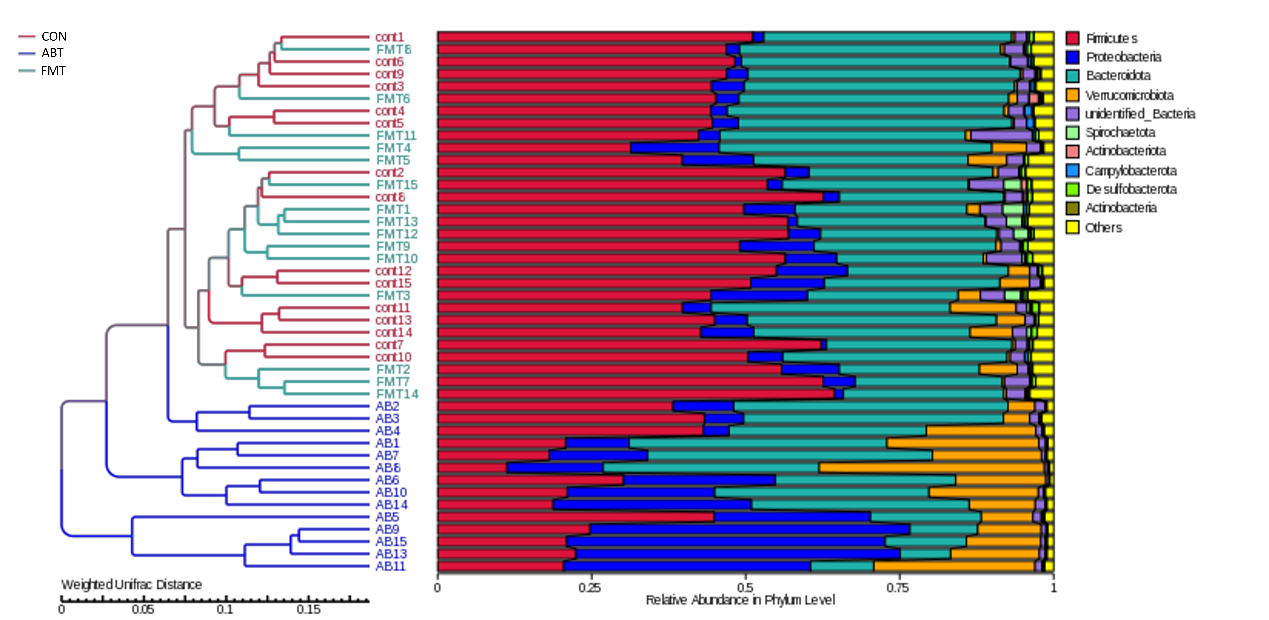


Fig S1. UPGMA clustering tree based on weighted Unifrac distance.


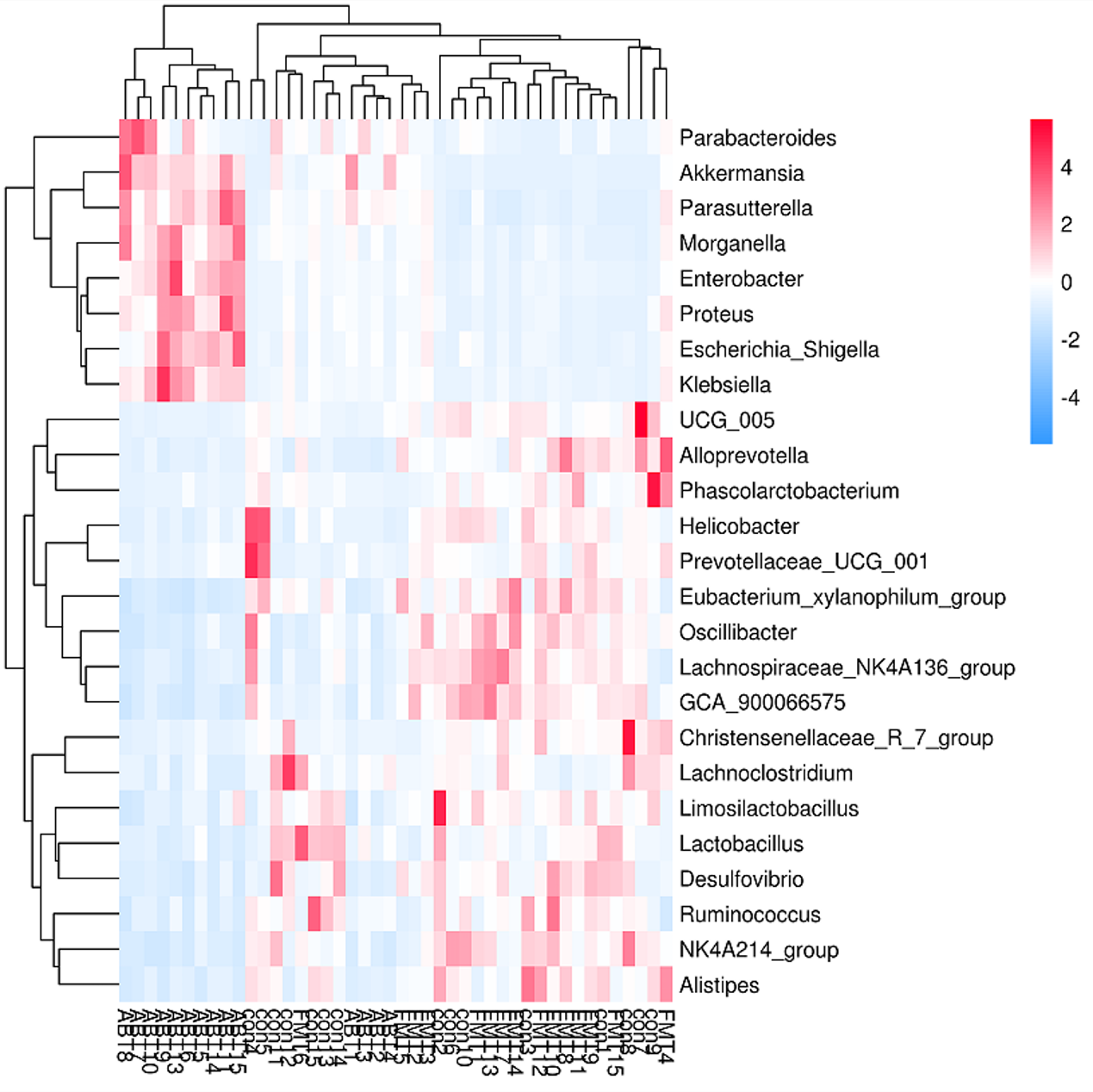


Fig S2. Clustering heatmap of relative abundance at the genus level


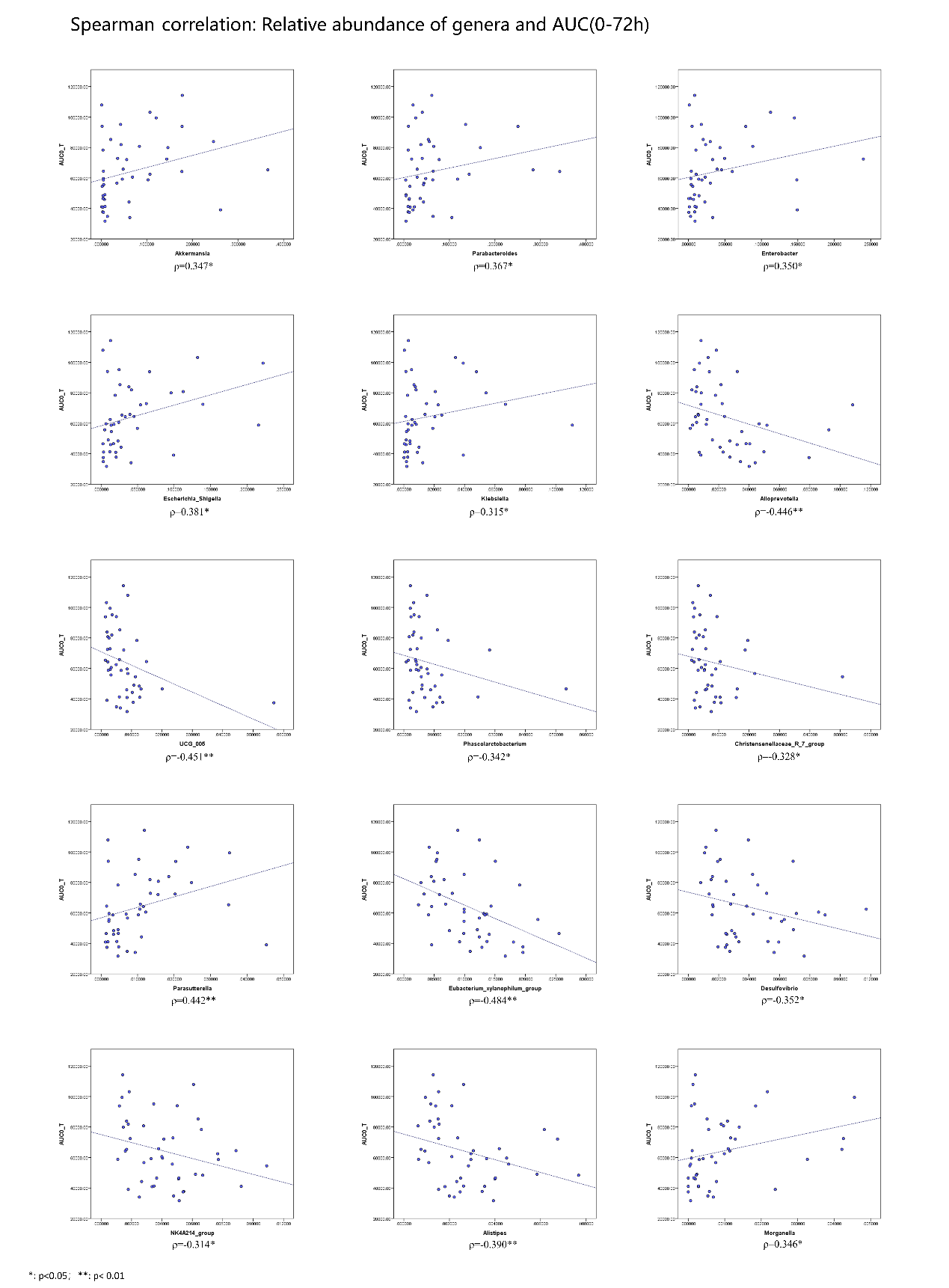


Fig S3. Plot of monotonic correlation analysis of AUC0-T and relative abundance of genera. ρ is Spearman correlation coefficient; * is p<0.05; ** is p<0.01.


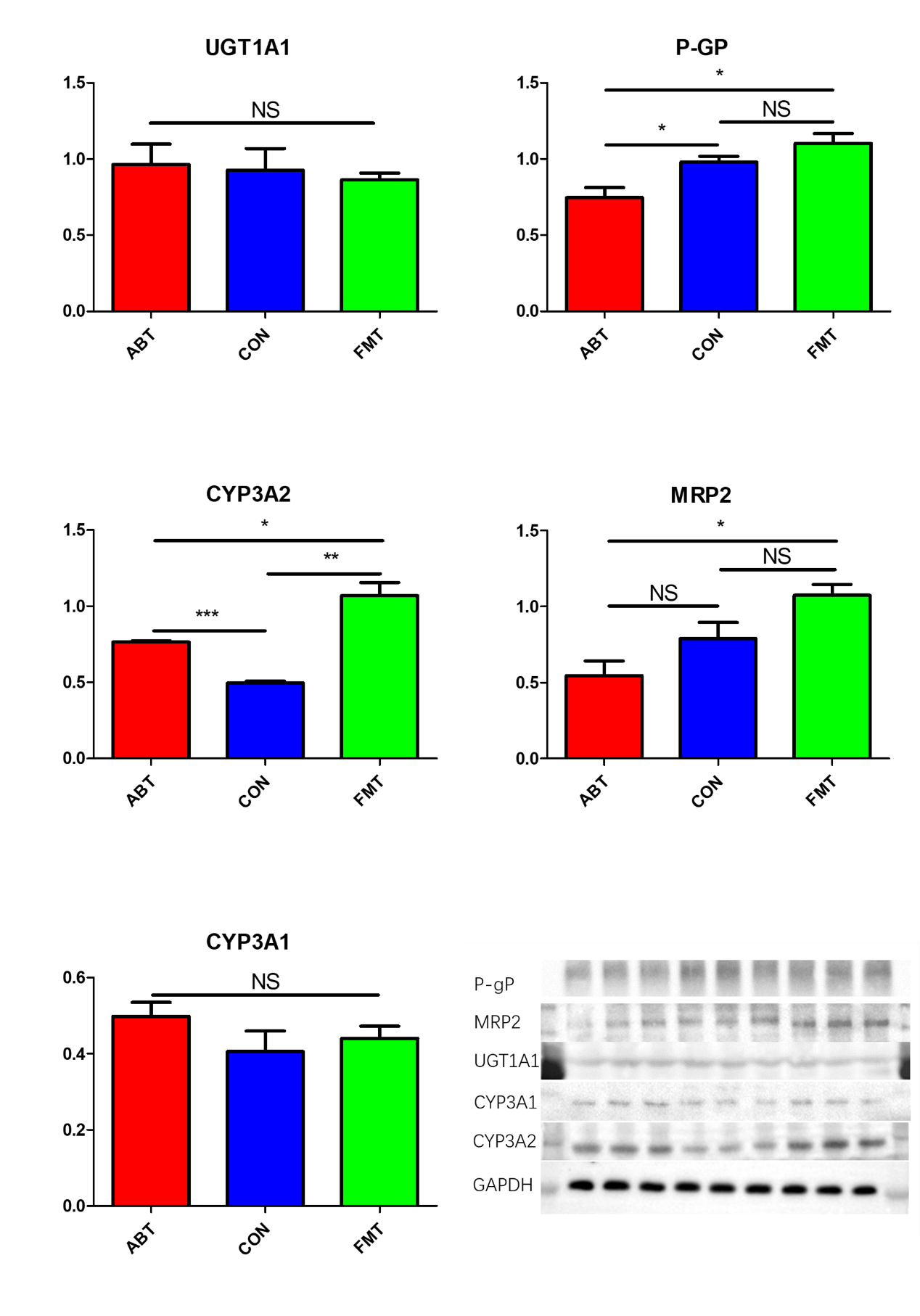


Fig. S4. Changes in protein expression of drug metabolic enzymes and drug transporters in intestine


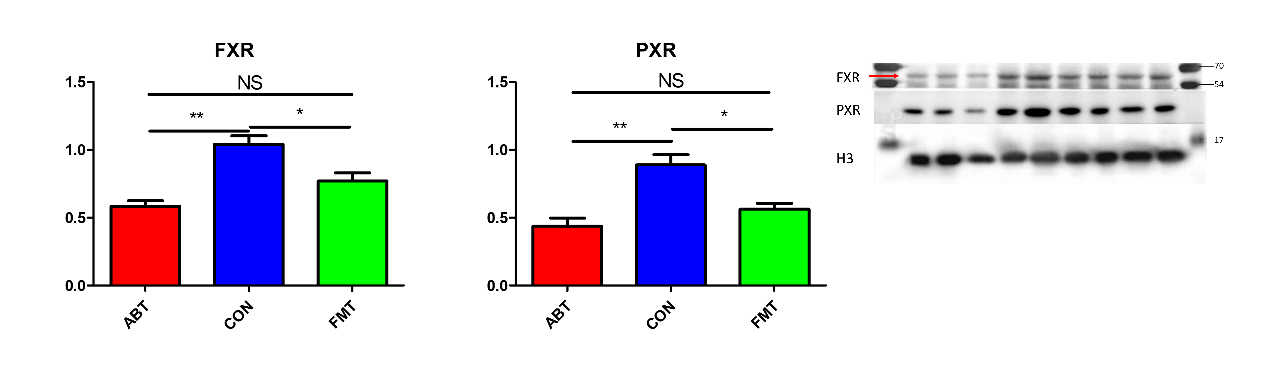


Fig. S5. Changes in protein expression of nuclear receptors FXR and PXR in liver
