# Supplementary figures and images for: Effects of intestinal microbiota on pharmacokinetics of cyclosporine a in rats
Source: Front Microbiol. 2022 Nov 22;13:1032290. doi: 10.3389/fmicb.2022.1032290 (PMC9723225; doi:10.3389/fmicb.2022.1032290)

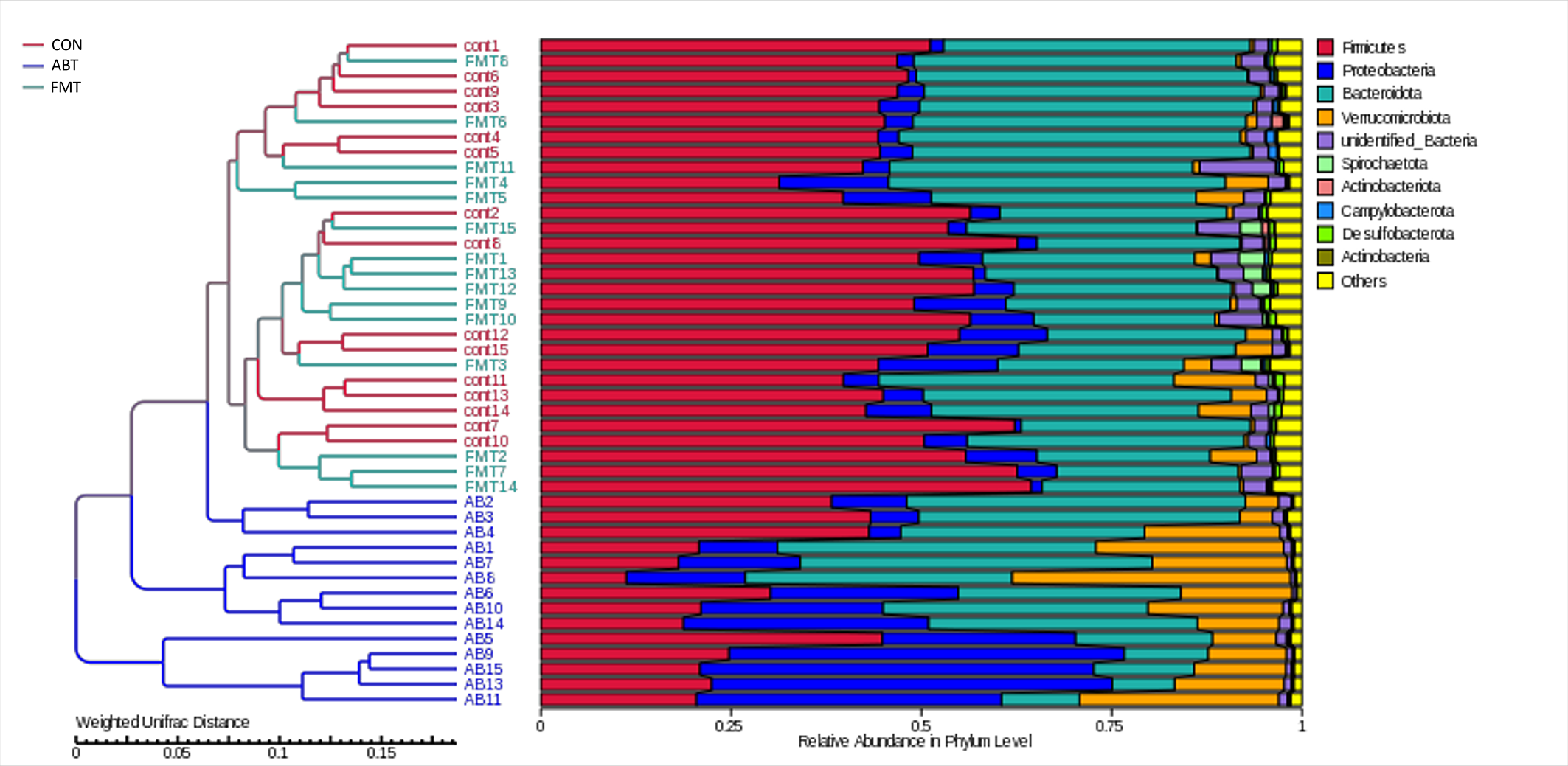

Supplement: Supplementary file 2 [file Image_1.TIF]

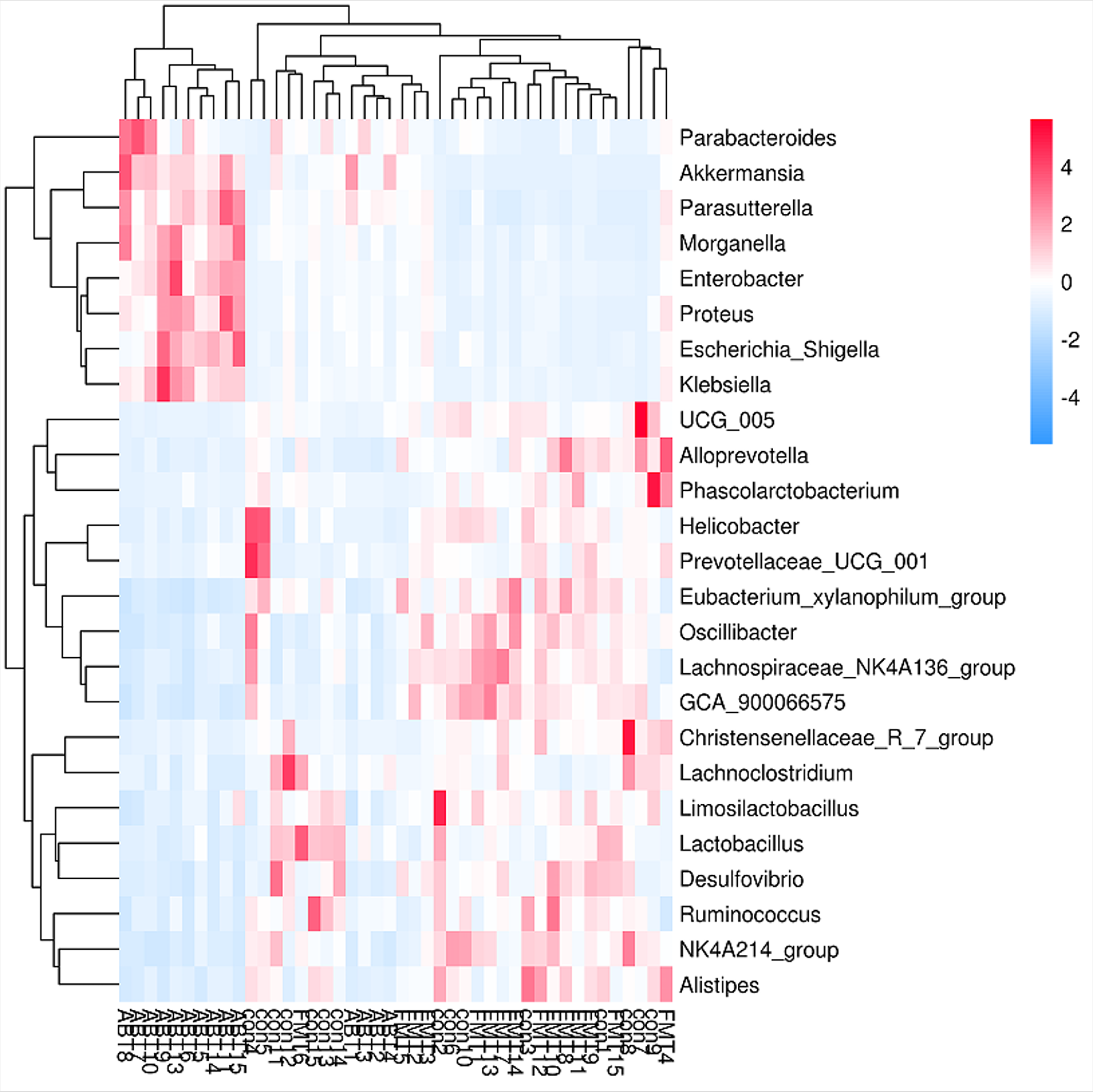

Supplement: Supplementary file 3 [file Image_2.TIF]

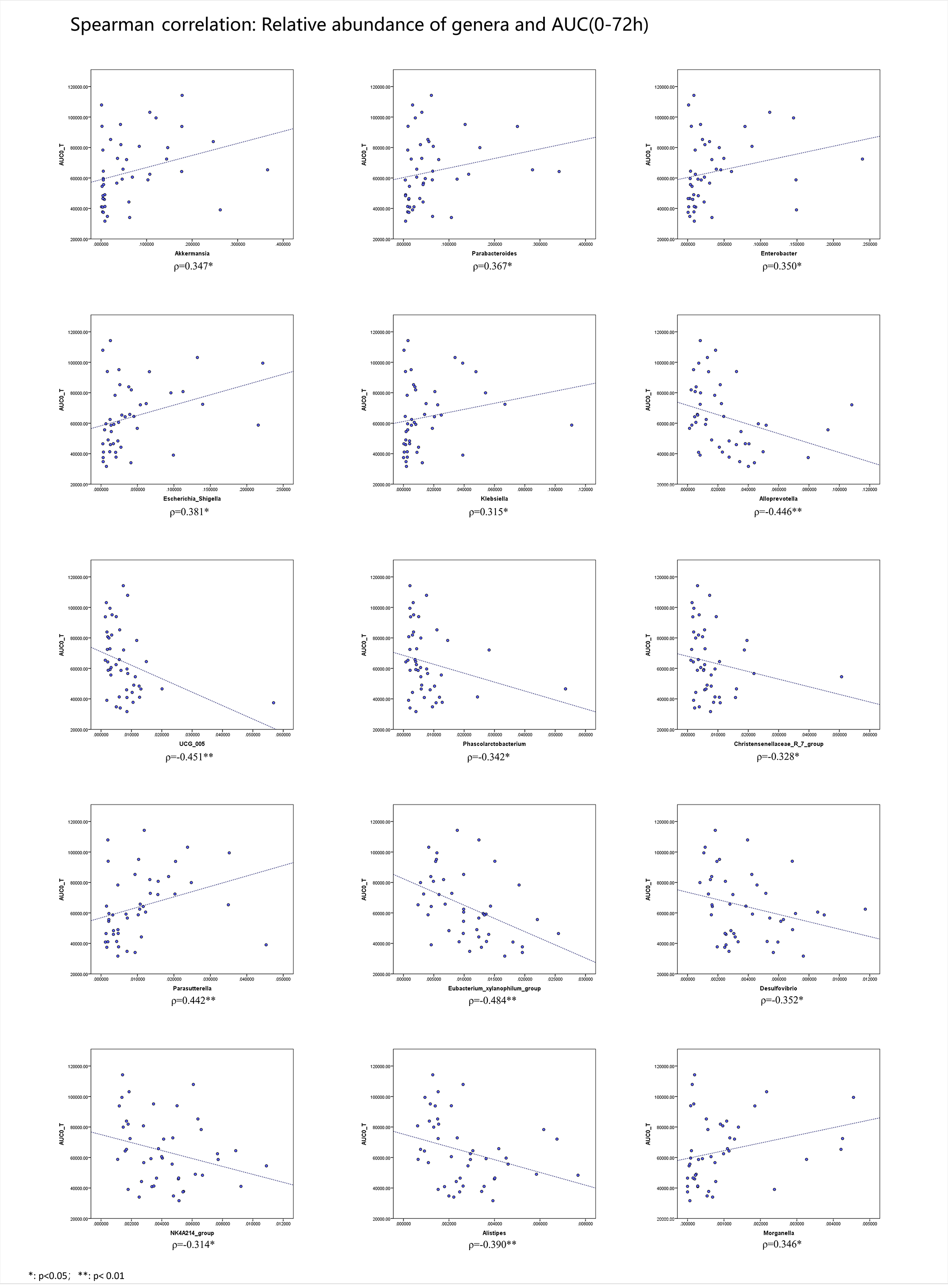

Supplement: Supplementary file 4 [file Image_3.TIF]

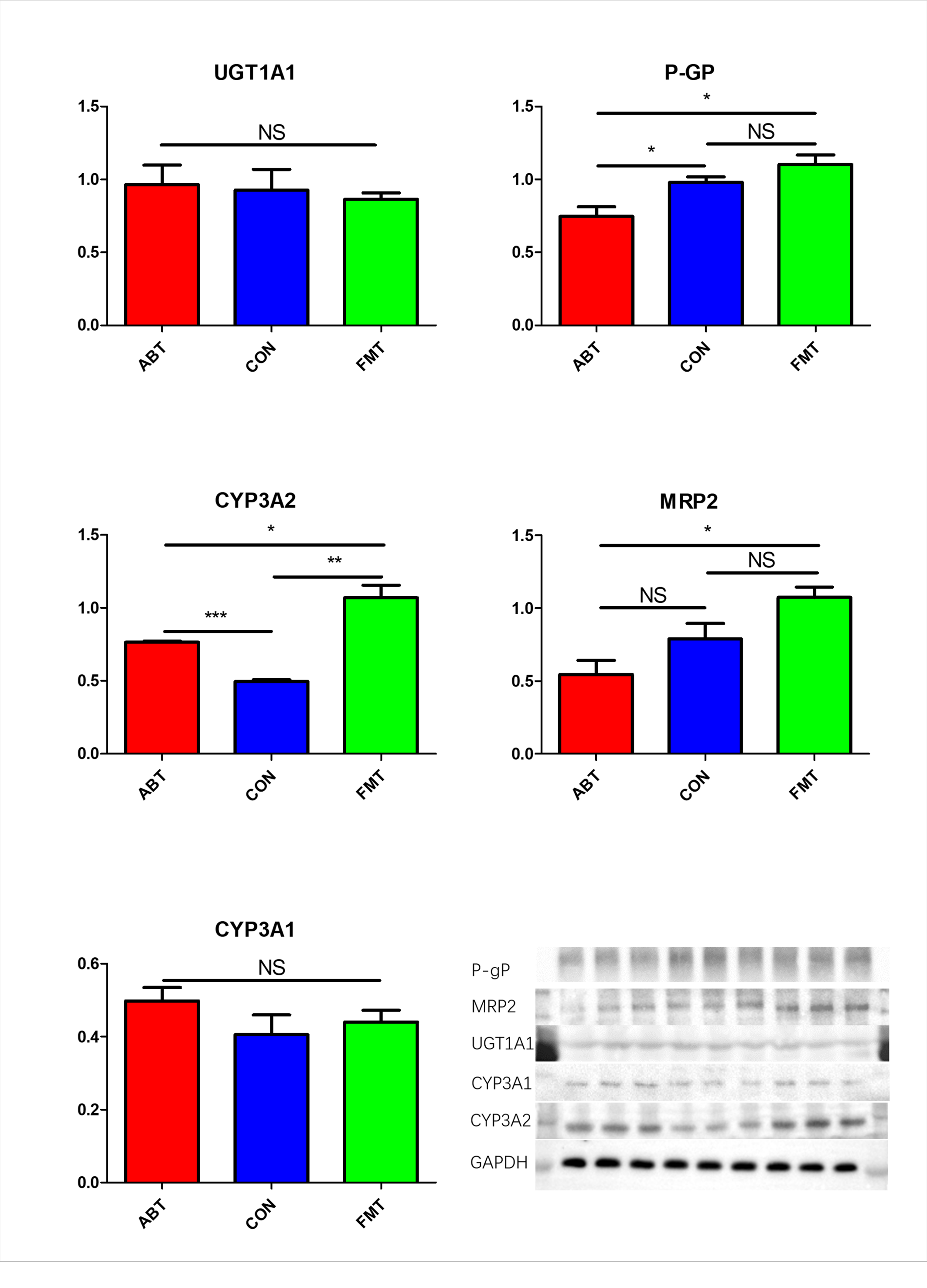

Supplement: Supplementary file 5 [file Image_4.TIF]

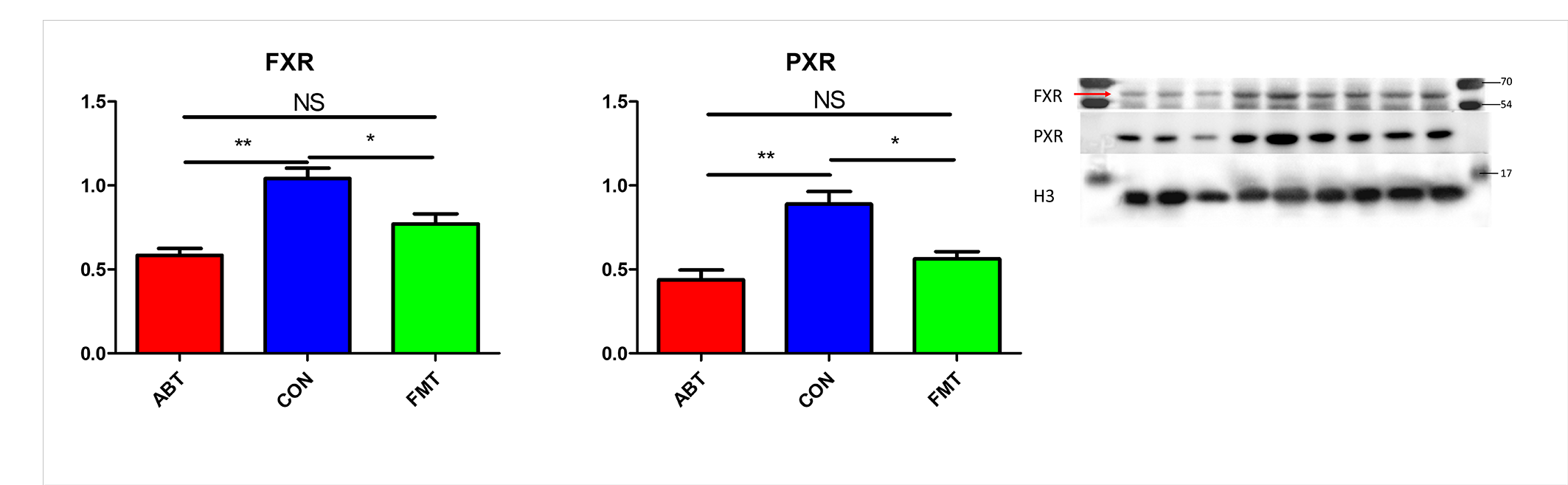

Supplement: Supplementary file 6 [file Image_5.TIF]
